# Associations between dog keeping and indoor dust microbiota

**DOI:** 10.1038/s41598-021-84790-w

**Published:** 2021-03-05

**Authors:** Jenni M. Mäki, Pirkka V. Kirjavainen, Martin Täubel, Eija Piippo-Savolainen, Katri Backman, Anne Hyvärinen, Pauli Tuoresmäki, Balamuralikrishna Jayaprakash, Joachim Heinrich, Gunda Herberth, Marie Standl, Juha Pekkanen, Anne M. Karvonen

**Affiliations:** 1 Department of Health Security, Finnish Institute for Health and Welfare, Neulaniementie 4, P.O. Box 95, 70701 Kuopio, Finland; 2 Department of Paediatrics, Kuopio University Hospital, P.O. Box 100, 70029 Kuopio, Finland; 3 Department of Clinical Nutrition, Institute of Public Health and Clinical Nutrition, University of Eastern Finland, Yliopistonranta 1, 70210 Kuopio, Finland; 4 University of Eastern Finland, P.O. Box 100, 70029 Kuopio, Finland; 5 Institute of Epidemiology, Helmholtz Zentrum München - German Research Center for Environmental Health, Ingolstädter Landstraße 1, 85764 Neuherberg, Germany; 6 Institute and Clinic for Occupational, Social and Environmental Medicine, University Hospital, LMU Munich, Ziemssenstraße 1, 80336 Munich, Germany; 7 Allergy and Lung Health Unit, Melbourne School of Population and Global Health, University of Melbourne, 207 Bouverie St, Carlton, Melbourne, VIC 3053 Australia; 8 Department of Environmental Immunology/Core Facility Studies, Helmholtz Centre for Environmental Research – UFZ, Permoserstraße 15, 04318 Leipzig, Germany; 9 Department of Public Health, University of Helsinki, P.O. Box 20, 00014 Helsinki, Finland

**Keywords:** Microbiology, Environmental sciences

## Abstract

Living with dogs appears to protect against allergic diseases and airway infections, an effect possibly linked with immunomodulation by microbial exposures associated with dogs. The aim of this study was to characterize the influence of dog ownership on house dust microbiota composition. The bacterial and fungal microbiota was characterized with Illumina MiSeq sequencing from floor dust samples collected from homes in a Finnish rural-suburban (LUKAS2, N = 182) birth cohort, and the results were replicated in a German urban (LISA, N = 284) birth cohort. Human associated bacteria variable was created by summing up the relative abundances of five bacterial taxa. Bacterial richness, Shannon index and the relative abundances of seven bacterial genera, mostly within the phyla *Proteobacteria* and *Firmicutes*, were significantly higher in the dog than in the non-dog homes, whereas the relative abundance of human associated bacteria was lower. The results were largely replicated in LISA. Fungal microbiota richness and abundance of *Leucosporidiella* genus were higher in dog homes in LUKAS2 and the latter association replicated in LISA. Our study confirms that dog ownership is reproducibly associated with increased bacterial richness and diversity in house dust and identifies specific dog ownership-associated genera. Dogs appeared to have more limited influence on the fungal than bacterial indoor microbiota.

## Introduction

Studies on dog ownership at early age as a risk or protective factor for asthma and allergy have shown inconsistent results^1, 2^. In the previous study cohort dog ownership and dog staying less indoors have been shown to be associated with less respiratory infections during the first year of life^3^. The reason for this protective association is unclear but the influence of dogs on the indoor microbiota could be one. Indoor microbial richness has been shown to be inversely associated with asthma^4^, however, its role on the other allergic diseases or respiratory infections have not yet been studied.

The occupants have the greatest influence in determining the composition of the indoor bacterial microbiome composition in a given household^5–7^. The occupants bring microbes from outdoors into the indoor environment, for example via their clothes and shoes and spread/shed microbes of their own microbiota (mostly from skin and oral cavity), which settle on floors and mattresses^8, 9^. Also dogs increase the bacterial diversity and the abundance of specific taxa^10, 11^, while the influence on the fungal microbiota seems less pronounced^5, 7^. One of the characteristic influence of dog ownership, that may be of health relevance ^12, 13^ is that it might reduce the relative abundance of human-related taxa^14^ in the indoor microbiota. As with the human occupants, dogs can influence the indoor microbiota by shedding microbes of their own microbiota as well as by carrying soil-, plant-, or elsewhere derived particulate matter with microbes from outdoors to indoors. The relative contribution of these dog-related mechanisms on the indoor microbiota is unclear^7, 15^.

Dog skin bacterial community includes partly the same bacteria than human skin microbiota^16^. Dog’s bacterial community varies across different body sites and the main phyla living on dog skin are Proteobacteria, Firmicutes, Bacteroidetes, Actinobacteria and Cyanobacteria^16, 17^. Human skin´s microbiota is diverse, but dominated by in particular *Staphylococci* (phylum Firmicutes), *Propionibacteria* (p. Actinobacteria), and *Corynebacteria* (p. Actinobacteria)^18^. The overlap between human and dog skin microbiota composition^16^ makes distinguishing the relative contribution of these two sources to indoor microbiota difficult. In addition to occupants and their dogs several other factors can have strong influence on the indoor microbiota composition. For example microbial richness is positively associated with farming^4, 19, 20^ and water leaks^21^ and inversely with urbanization^21, 22^, while cat ownership seem to have little influence^5, 11, 15, 23^.

The aim of this study was to determine whether dog ownership is associated with consistent characteristics in bacterial and fungal house dust microbiota, such as higher diversity compared to non-dog homes, decrease of the relative proportion of human-related taxa or increased abundance of dog or soil associated taxa. To answer this aim we utilized two different study populations located in two European countries.

## Results

The present study population was comprised of LUKAS2 and LISA birth cohorts. There were 56 (31%) dog homes and 126 (69%) non-dog homes in LUKAS2 cohort and respective figures were 18 (6%) and 266 (94%) in LISA cohort (Additional Table S1). Dust samples were collected throughout the year. In LUKAS2, 54 (30%) samples were collected during snow cover (29% of dog homes and 30% of non-dog homes) and 128 samples during no snow cover.

### Richness and Shannon entropy (alpha diversity)

**Figure 1:**
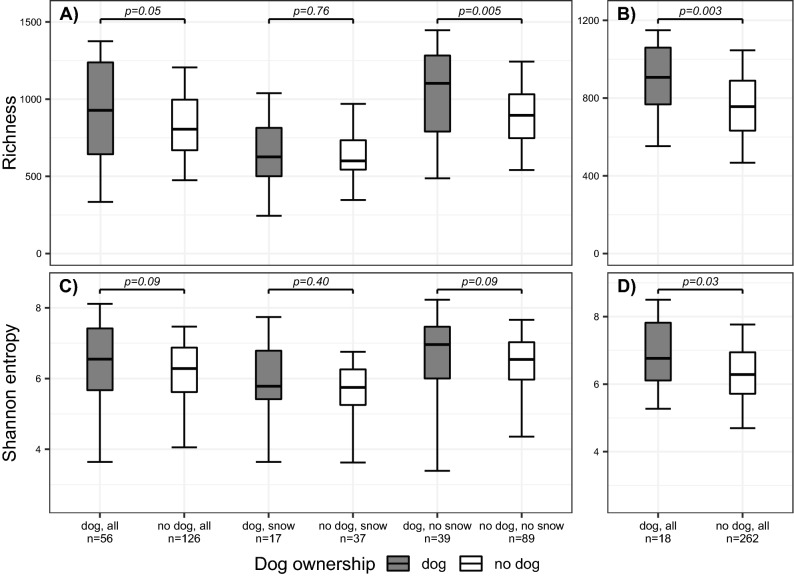
Box-plots of bacterial richness and Shannon entropy in dog and non-dog homes in two cohorts. Box-plots of bacterial richness (Chao1) in (**A**) LUKAS2 and (**B**) LISA, and Shannon entropy in (**C**) LUKAS2 and (**D**) LISA in house dust samples collected from dog homes (grey) and non-dog homes (white). In LUKAS2, additional comparison is made by samples taken during snow cover (snow) and without snow cover (no snow). *p*-values are from Mann–Whitney U-test in LUKAS2 and Weighted Mann–Whitney U-test in LISA. The boxplots present 5^th^ percentile, first quartile, median, third quartile, and 95^th^ percentile of the values.

Bacterial richness (Chao1) was significantly higher in the dog homes than in the non-dog homes in LUKAS and LISA (Fig. 1A, B, respectively*)*. In analysis stratified by snow cover bacterial richness was significantly higher in the dog homes only when the ground was not covered by snow (Fig. 1A). Bacterial Shannon entropy was higher in the dog homes than non-dog homes in both cohorts, although in LUKAS2 only when the ground was covered by snow, and even then the difference was not statistical significant (Fig. 1C, D in LUKAS and LISA*,* respectively).

**Figure 2:**
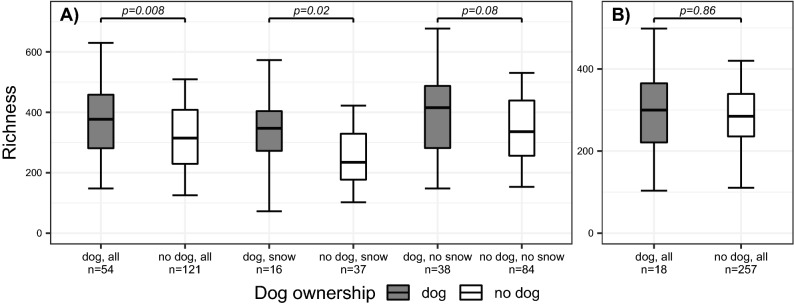
Box-plots of fungal richness in dust samples from dog and non-dog homes in two cohorts. Box-plots of fungal richness (Chao1) in house dust samples collected from dog homes (grey) and non-dog homes (white) in (**A**) LUKAS2 and (**B**) LISA cohorts. In LUKAS2, additional comparison is made by samples taken during snow cover (snow) and without snow cover (no snow). *p*-values are from Mann–Whitney U-test in LUKA S2 and Weighted Mann–Whitney U-test in LISA. The boxplots present 5^th^ percentile, first quartile, median, third quartile, and 95^th^ percentile of the values.

Fungal richness (Chao1) was higher in the dog homes in LUKAS2, but not in LISA (Fig. 2A, B, respectively). In LUKAS2, the difference was significant only when the ground was covered by snow (Fig. 2A). No such differences were observed for fungal Shannon entropy in either cohorts (Supplemental Fig. S1A, B).

### Compositional dissimilarity between samples (beta-diversity)

**Figure 3:**
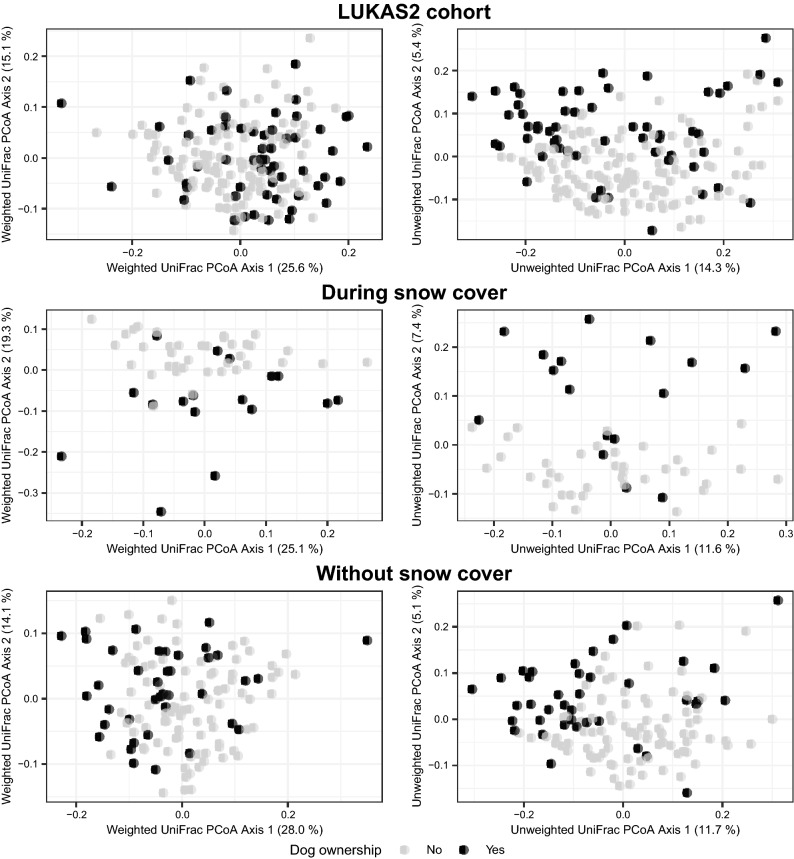
Plots of two bacterial axes scores by dog houses stratified by snow cover in LUKAS2. Plots of the first (PCoA1) and the second (PCoA2) axes scores from bacterial weighted and unweighted UniFrac based Principal Coordinate Analyses in LUKAS2 cohort by houses with (black dots) or without (grey dots) dog(s). Percentages of the variance explained by the axis scores are in the parentheses. Top two plots were made using dust samples from the whole LUKAS2 cohort (excl. farmers), two middle plots with samples collected without snow cover, and two plots in the bottom with samples when snow was on the ground.

The bacterial microbiota in the dog homes had compositional presence-absence and relative abundance characteristics that distinguished them from the non-dog homes in LUKAS2 (Fig. 3).

Overall, the dog ownership explained 3% of the total variance in the presence-absence and 2% of that in the relative abundance patterns as indicated by unweighted and weighted UniFrac analyses, respectively (*p* = *0.0002* in both). Similar PCoA plots without statistical tests for data in LISA are shown in the Additional material (Fig. S2).

The dog homes had also compositional fungal presence-absence but not relative abundance characteristics that distinguished them from the non-dog homes as indicated by the Binary Jaccard (*p* = *0.002*) and Bray Curtis distance metrics analyses (*p* = *0.20)*, respectively*.* PCoA plots in LUKAS2 and LISA cohorts are shown in the Additional material (Figs. S3 and S4, respectively).

### Bacterial taxa associated with the dog-keeping

In LUKAS2, the relative abundances of 55 bacterial taxa (from phylum to genera) were different between the dog and non-dog homes with correction for multiple testing (Additional material Table S2). Of these taxa, 19 were assigned genera (Additional material Table S2), of which 7 were more abundant in the dog homes and most of them were from Proteobacteria and Firmicutes phyla (Table 1). These were genera within Firmicutes: *Clostridium*, *Megamonas*; genera within Proteobacteria: *Lampropedia*, *Conchiformibius*, *Helicobacter*, *Pasteurella;* and *Mycoplasma* (p. Tenericutes) (Table 1). Six of these seven genera (all except the *Lamprobedia* genus), had higher abundance in the dog than non-dog homes also in the LISA (Table 1)*.*

**Table 1:** Comparisons between relative abundances of seven bacterial and one fungal genera in households with or without dog in LUKAS2 and LISA birth cohort studies.

Taxonomy (*Phylum; Class; Order; Family; Genus*)	LUKAS2					LISA				
	Dog		No dog		*p*	Dog		No dog		*p*
	< DL	med. (%)	< DL	med. (%)		< DL	med.	< DL	med.	
**Bacteria**										
*Firmicutes; Clostridia; Clostridiales; Clostridiaceae; Clostridium*	1.8	0.24	1.6	0.08	< *0.001*	0.0	0.52%	0.0	0.16%	*0.01*
*Firmicutes; Clostridia; Clostridiales; Veillonellaceae; Megamonas*	26.8	0.06	73.0	0	< *0.001*	11.1	0.11%	44.7	0.01%	< *0.001*
*Proteobacteria; Betaproteobacteria; Burkholderiales; Comamonadaceae; Lampropedia*	51.8	0	93.7	0	< *0.001*	100.0	–	100.0	–	–
*Proteobacteria; Betaproteobacteria; Neisseriales; Neisseriaceae; Conchiformibius*	26.8	0.07	75.4	0	< *0.001*	33.3	0.01%	98.1	0%	< *0.001*
*Proteobacteria; Epsilonproteobacteria; Campylobacterales; Helicobacteraceae; Helicobacter*	51.8	0	87.3	0	< *0.001*	55.6	0%	93.5	0%	*0.05*
*Proteobacteria; Gammaproteobacteria; Pasteurellales; Pasteurellaceae; Pasteurella*	37.5	0.02	91.3	0	< *0.001*	61.1	0%	96.6	0%	*0.01*
*Tenericutes; Mollicutes; Mycoplasmatales; Mycoplasmataceae; Mycoplasma*	50.0	0.003	82.5	0	< *0.001*	66.7	0%	97.3	0%	*0.04*
**Fungi**										
*Basidiomycota; Microbotryomycetes; Leucosporidiales; Leucosporidiaceae; Leucosporidiella*	7.4	0.10	28.1	0.03	*0.0005*	22.2	0.05%	32.7	0.02%	*0.04*

< DL percentage of samples under detection limit; med. median relative abundance; – not found in LISA. The number of dog homes (the number of no dog homes) in bacterial data in LUKAS2 and LISA: 56 (126) and 18 (262). The number of dog homes (the number of no dog homes) in fungal data in LUKAS2 and LISA: 54 (121) and 18 (257). *p*-values are from Mann–Whitney U-test in LUKAS2 and weighted Mann–Whitney U-test in LISA.

**Figure 4:**
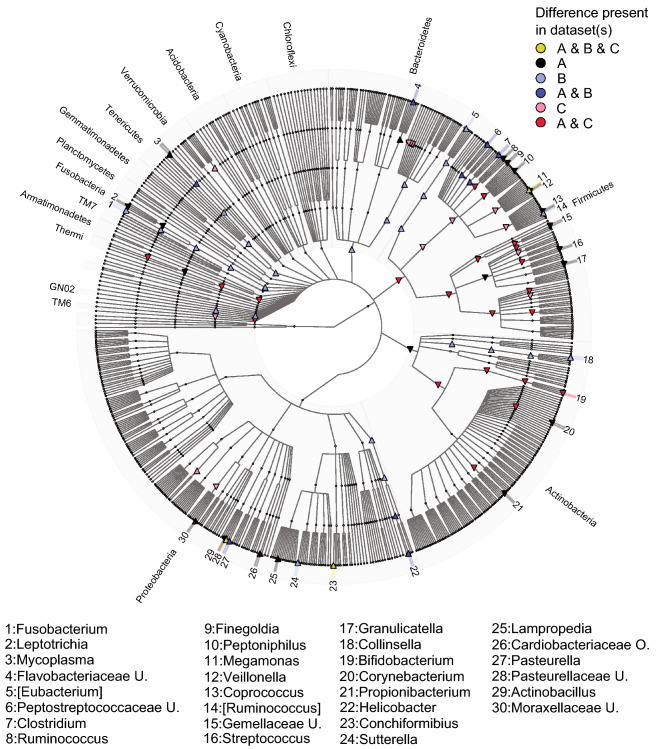
Bacterial taxa associated with dog and non-dog homes in LUKAS2 cohort, determined by ANCOM. Cladogram is additionally stratified by the sampling time: snow cover and no-snow cover. From the highest rank to the lowest, the major taxonomic ranks are domain (in the middle), phylum, class, order, family and genus (in the farthest layer). Genera that differ between the dog homes and non-dog homes are numbered in the figure. U. denotes “unassigned”, O. “other” genus within a family, and brackets indicate candidate taxonomy. Three datasets were created [(**A**) the whole cohort, (**B**) only samples, when snow was on the ground, and (**C**) only samples without snow on the ground] for this phylogenetic tree. If the top of triangle is pointing up, the relative abundance of the taxa is higher in dog homes. If the top of triangle is pointing down, the relative abundance of the taxa is lower in dog homes than non-dog homes. Yellow = the difference is found in all three datasets; black = the difference is seen only when the whole cohort is used (dataset **A**), but the difference disappears when data stratified by snow cover; light blue = the difference is only found when snow is on the ground (dataset **B**); dark blue = the difference is found in the whole cohort and when snow was on the ground (datasets **A** and **B**); light red = the difference is seen only without snow cover (dataset **C**); dark red = the difference is seen with the whole cohort and without snow cover (datasets **A** and **C**), but not when snow cover.

The relative abundances of 10 assigned genera were higher in the dog than non-dog homes in the whole LUKAS2 cohort and when samples were collected while snow covered the ground (Fig. 4, dark and light blue triangles). Of these, 5 were more abundant in the dog than non-dog homes only when snow covered the ground (Fig. 4, light blue triangles): *Collinsella*, [*Ruminococcus*]*,* [*Eubacterium*], *Fusobacterium* and *Sutterella* (Additional Table S3). No differences were observed when samples collected without snow cover were analyzed in LUKAS2, nor in the whole LISA (Additional Table S3).

### Fungal taxa associated with the dog-keeping

Only 5 fungal taxa, including two genera (*Arthrinium* and *Leucosporidiella*), were associated with dog ownership in LUKAS2 after correcting for multiple testing (Additional Table S4). Of these two, only the genus *Leucosporidiella* was more abundant in the dog than non-dog homes, and this result replicated in LISA (Table 1). In addition, the relative abundance of *Udeniomyces* was higher in the dog than non-dog homes in LUKAS2 when samples were collected while snow covered the ground (Additional Table S3).

### Relative abundance of human-associated bacteria

The relative abundance of human-associated bacteria, as indicated by HSP (“human source proxy”, a sum variable of human-associated bacterial taxa derived from sequencing data), was lower in the dog than non-dog homes in LUKAS2 (median relative abundance 13.7% vs. 22.4%, *p* < *0.0001*, respectively). Human-associated bacteria were also more abundant in non-dog homes in the LISA cohort (median 29.7% vs. 41.4%, *p* = *0.15,* respectively). Snow cover did not influence the association between the dog ownership and HSP in LUKAS2 (with snow cover 14.0% vs. 25.3%, *p* = *0.0014* and without snow cover 13.4% vs. 22.0%, *p* = *0.0003*).

### Differences in bacterial and fungal levels in the dog versus non-dog homes (qPCR)

In LUKAS2, the concentrations of Gram-negative bacteria in house dust (cell equivalent per mg of dust) were higher and those of Gram-positive bacteria lower in the dog than non-dog homes (Additional Fig. S5B, C). The higher concentrations of Gram-negative bacteria in the dog than non-dog homes was more pronounced during snow cover and the lower level of Gram-positive bacteria during no snow cover (Additional Fig. S6B, C). Similar results were observed when considering bacterial loads (cell equivalent per m^2^ floor area) rather than concentrations (Additional Fig. S7B, C). The concentration or the loads of total bacteria (sum of Gram-positive and Gram-negative) did not differ between the dog and non-dog homes (Additional Fig. S5A, D). No differences were found between the dog homes and non-dog homes in the concentrations or loads of total fungi, and snow cover did not affect the results (data not shown).

### Correlations between bacterial and fungal diversity indices, genera and qPCRs in LUKAS2

The highest correlations were found between bacterial richness and Shannon entropy, and between Gram-negative or Gram-positive bacteria and total bacteria (*r* > *0.80*, Additional Fig. S8). Fungal richness correlated with bacterial richness (*r* = *0.62*). The correlation coefficients between dog-associated bacterial genera varied mostly between *0.30* and *0.60.* HSP correlated weakly and negatively with bacterial richness and Shannon entropy (*r* =  − *0.18* and − *0.16*, respectively, *p* < *0.05*) and the seven consistently dog-associated genera (listed in Table 1) (− *0.20* and − *0.34, p* < *0.05*) (Additional Fig. S8). No correlations were observed between HSP and the five dog-associated bacterial genera that were specifically associated with the dog ownership during snow cover on the ground (*p* > *0.1*).

## Discussion

We show in here that the dogs in the homes of small children have characteristic influences on the indoor bacterial and to lesser extent on fungal microbiota that are consistent between two study populations. Dog ownership was positively associated with bacterial richness and diversity in house dust, the latter taking into account also the balance of relative abundances of the detected taxa. The bacterial genera *Clostridium, Megamonas, Conchiformibius, Helicobacter, Pasteurella* and *Mycoplasma* and the fungal genus *Leucosporidiella* were positively associated with dog keeping, while the relative abundance of human-associated bacteria decreased*.*

The increased diversity of bacterial microbiota in the dog homes is intuitive and in line with earlier studies^5, 15^. The dog ownership associated bacteria in indoor dust can be sourced directly from the dog’s own microbiota or from the outdoor environment with the dog acting as a vector^12, 13^. This could indicate that the dog-keeping associated increase in richness is more due to bacteria carried in by the dogs from the outdoor environment than from the dogs’ own microbiota^5, 11^. This is supported by our finding that the richness was not higher in the dog homes when there was snow cover and the dogs were thus less likely to carry soil indoors. Other studies, including a previous Finnish study, have suggested that the taxa increased in homes with dogs derive directly from the dog’s own microbiota and transfer of microbes by pets might not be very considerable^22^. Moreover, a previous study in LISA showed that the residential greenspaces around the home did not influence dog ownership associated increase in home endotoxin levels. So while our findings in the analyses stratified by snow cover indicate that bacterial diversity and especially the species richness depend on the surrounding environment of the house, quantitatively the surroundings have less clear influence on the dog ownership associated influences on the home microbiota^24^, which is supported by presented qPCR results in the present study.

Notably, all of the seven bacterial genera that were consistently associated with dog keeping could originate from the dog’s own microbiota. Genus *Megamonas* is found in the dog’s gastrointestinal tract^25^, perianal region, and in lower abundance also from other body sites such as dorsal back or nasal skin^17^. *Conchiformibius* is part of the dog-microbiota and has been identified from the dog’s oral cavity, nasal skin, chin and inner pinna^17^. Genus *Pasteurella* has been identified especially from the dog’s inner pinna and chin, also in smaller amounts from another body sites, such as dorsal back^17^. *Mycoplasma* has been detected in the dog’s oral cavity^26^. Source allocation of genus level taxa is, however, in many cases not straight forward and also humans or other animals could have acted as sources for some of these genera. For example, the genera *Megamonas* and *Clostridium* may originate from human gastrointestinal tract^27, 28^.

During snow cover microbial levels in outdoor air are lower^29^ and less microbes are likely to be transferred indoors through ventilation and on the surface of human occupants (clothing) and their pets. With the less ‘noise’ caused by the fluctuating presence of outdoor microbes in indoor dust, sampling during snow cover may result in greater sensitivity to detect indoor microbes associated with the dog microbiota. Indeed, five additional dog-associated bacterial genera were detected exclusively during snow cover in the Finnish cohort. In contrast, the level of human-associated bacterial taxa did not differ between samples collected during snow cover and without snow cover. This is against the notion of less ‘noise’ during snow cover but as the dominant source of indoor microbes the levels of human-associated bacteria may be more consistent and with their abundance stand out through the noise.

One of the intuitive characteristic influences of dog ownership was that the relative abundance of human-associated bacteria in floor dust was decreased as much as over one third, independently of the snow cover. This effect may be of health relevance as the high relative abundance of human associated microbes potentially translates to higher relative abundance of human pathogens and immunological danger signals with negative immunomodulatory effects that may predispose to development of asthma in later life^12, 13, 30^. Future studies are warranted to investigate whether this reduction in relative abundance of human-associated bacteria and/or the increase in dog-associated bacteria may explain the reduced risk of respiratory infections and asthma in children growing up in homes with pet(s)^31^.

Fungal richness was significantly higher in the dog compared to the non-dog homes only in the Finnish house dust samples, when snow cover was present during sampling. As discussed above, during no snow cover, the outdoor environmental sources might mask the influence of dog on the indoor microbiota and thus be less sensitive period to study the dog effect. The limited influence of dogs on fungal diversity is supported by a previous study^5^. The only fungal genus, which was consistently increased with the dog ownership, *Leucosporidiales,* is a yeast that is known to occur in water and in soil and thus maybe be something typically carried indoors by the dog after outdoor environmental contact^32, 33^. Overall, much of the fungal content found in homes originates from the outdoors and the community structure is mostly characteristic to the respective climate and geographical region^6, 7, 34^. Unlike for bacteria, the dog itself is not a strong source of fungal taxa in house dust, and its role as a vehicle for transfer of outdoor microbes indoors appears to be less relevant for fungi.

The obvious strength of this study was the inclusion of two different studies representing different countries and study populations, located in rural and semi-urban Finnish and urban German environment. The results replicated very well in LISA study, though the dust collection had distinctiveness between cohorts (for example samples were taken from living room vs. bedroom floors, nylon dust socks vs. ALK filters, and they had different storage time in freeze). However, we cannot exclude the possibility that even more similarities would have been found with identical sampling. The amount of dog households was considerably greater in the Finnish cohort probably due to the fact that Finnish data was collected in a more suburban environment whereas the German cohort was performed in an urban environment. Also, in the Finnish study, we had the possibility to analyze house dust during snow and during no snow cover, which enables sensitivity analysis with or without limited influence of microbial carriage from outdoors. One limitation of our study is that our taxa difference was based on relative abundances of taxa, not presence/absence, which might be a reason why we missed some genera compared to earlier studies. Another limitation is that we had no specification on dog breed, fur characteristics (length, thickness). Also the environment of daily walks was undetermined (asphalt, forest trail, etc.). The results should be confirmed in future studies in different environments.

## Conclusions

Our study confirms that dog ownership is reproducibly associated with increased bacterial richness and diversity in house dust and identifies specific dog ownership-associated genera. Dog indoors decreases the relative abundance of human associated bacteria. Dogs appeared to have more limited influence on the fungal than bacterial indoor microbiota.

## Methods

### LUKAS2 birth cohort study

The Finnish population based birth cohort, LUKAS2 (n = 182), was recruited between May 2004 and May 2005. All pregnant women, who planned to give their birth in the Kuopio University hospital, were invited to join the study. The inclusion and exclusion criteria have been described earlier in detail^35^. In this study, all families lived in single family homes, semi-detached, or raw houses. From the present analyses, 9 farmers were excluded because of the strong influence of farming on indoor microbiota and in order to keep the main focus on suburban or rural families.

The first questionnaire was administered during the third trimester of pregnancy and follow-up data was collected from the mothers by self-administered questionnaires when the children were 2 months of age. Family and home characteristics were enquired in the first questionnaire and dog ownership in the 2-month questionnaire. The ethical permission of the study was granted by the Research Ethics Committee of the Hospital District of Northern Savo, Kuopio, Finland. Written consents were acquired from the parents of the participating children^35^. All methods were carried out in accordance with relevant guidelines and regulations in the manuscript. The total study populations in the present bacterial and fungal analyses were 182 (56 dog household and 126 no dog household) and 175 (54 dog household and 121 no dog household), respectively.

### LISA birth cohort study

The LISA study is a German population based birth cohort where neonates were recruited at birth in Munich, Wesel, Leipzig and Bad Honnef between 1997 and 1999 (n = 3094)^36^. The original study protocol was approved by the local ethics committee (the Bavarian Board of Physicians, reference number 01212). Written consents were acquired from the parents of the participating children. All methods were carried out in accordance with relevant guidelines and regulations in the manuscript.

Questionnaires contained information about sociodemographic factors, environmental exposures and health. The first questionnaire was administered to the parents at their child’s birth^36^. In the current study, we used a subset of the original study cohort, which has been described previously^37^. Shortly, a random sample of 250 participants and enrichment of all the rest asthma ever and ADHD cases (n = 114), were selected from the Leipzig and Munich study centers. Due to lack of samples (n = 51), unidentified sample IDs (n = 20), low amount of dust < 5 mg (n = 32), sample lost during sequencing (n = 2) and samples below the cut-off value of rarefaction curve in bacterial (n = 5) and fungal samples (n = 9), and excluding two farmers, the total study populations in the current bacterial and fungal analyses were 280 (18 dog household and 262 non-dog household) and 275 (18 dog household and 257 non-dog household), respectively.

### Dust samples

Living room floor dust samples were collected at 2 months of age in LUKAS2. The sampling was performed using a nylon sampling sock and by vacuuming an area of 1 m^2^ from a rug for two minutes or an area of 4 m^2^ from smooth floor for two minutes. Dust samples were homogenized by sieving through sterile strainer (pore size approx. 1 × 1 mm), dried in desiccator at 4 °C and then stored at − 20 °C in darkness until DNA extraction.

In the LISA study, dust samples were collected from bedroom floors when the children were 3 months of age^38^. The sample was collected using a vacuum cleaner supplied with an ALK sampling devices (ALK allergen mouthpiece; ALK, Hørsholm, Denmark) and stored at − 20°C^37^ post sampling at the study center in Germany and shipped for subsequent processing at the Finnish Institute for Health and Welfare (Kuopio, Finland). The dust was removed from the filters, size homogenized using sterile strainer as in LUKAS, aliquoted and stored at − 20 °C in darkness until DNA extraction^37^.

### Season of dust sampling, snow cover

Seasons of dust sampling were based on the date of house dust sampling and categorized in both study cohorts as follows: winter between December-February, spring March–May, summer June–August and autumn September–November. When the LUKAS2 children of the Finnish cohort were born, the study area was covered by snow from December 15^th^ 2004 until the end of March 2005. During winter, the temperature is below 0 °C, the ground is covered with snow and there is no visible soil. Thermic spring started in our study area March 31^st^ in 2005 and thermic spring lasted from six to nine weeks^39^. During the thermic spring the average daily temperature is between 0 and 10 °C. Snow melts from the open ground during two to three weeks and from forest two weeks later, on average, from the beginning of the thermic spring. Since flooring and in particular rugs are dust reservoirs and samples represent dust accumulated over time, we decided to use a two weeks lag-period after snow started covering the ground before categorizing the sampling done during snow cover (that is, from 1^st^ January 2004 to 31^st^ March 2005). According to this information, some of the analyses with LUKAS2 data were stratified by snow on the ground. Permanent snow cover does usually not exist in the German study centers of Leipzig and Munich, and thus, such snow cover variable was not created for LISA.

### DNA extraction, 16S rRNA gene and ITS PCR and amplicon sequencing

The protocols for DNA extraction, bacterial 16S rRNA gene and fungal ITS PCR and library preparation, amplicon sequencing and sequence processing have been described in detail earlier (for more detail, see ref.^12, 30, 37^). Further detail on the sample processing is also provided in the Supplemental Material. In brief, DNA extraction was carried out from a target amount of 20 mg of dust including a bead milling step and clean-up with Chemagic DNA Plant–kit (PerkinElmer chemagen Technologie GmbH, Germany) on a KingFisher DNA extraction robot. DNA was shipped frozen to a commercial sequencing partner (LGC Genomics, Germany). The bacterial 16S rRNA gene V4 region was amplified using 515F/806R primers^40^ and fungal ITS region by ITS1F/ITS2 primers^41^. These DNA amplicons were sequenced as 300 base pair paired-ends reads with Illumina MiSeq V3 Chemistry. Subsequent processing and analysis steps relied largely on QIIME (Quantitative Insights Into Microbial Ecology)^42^; software version 1.9.1. Sequences were clustered into operational taxonomic units (OTUs) at 97% similarity using the open-reference OTU-picking approach. Chloroplast and mitochondrial sequences were removed from the OTU tables. Alpha diversity indices Chao1 and Shannon were calculated with QIIME. Chao1 is an estimator of OTU (~ species) richness in a sample (i.e. the number of different OTUs), while the Shannon accounts for both the number of different OTUs and evenness in their relative abundance distribution in a sample. For the analysis of individual fungal taxa, taxonomic classification was done using FHiTINGS (Fungal High throughput Taxonomy Identification in NGS), calculating taxa-based OTU groups instead of clustering^43^. Negative (reagents) and positive (bacterial and fungal mock community) controls were included in the DNA extraction alongside processing of the actual house dust samples. The bacterial mock community consisted of equal concentration 1E + 8 cells/ml of seven species, while the fungal mock community was an assemblage of 43 species at either 1E + 4 or 1E + 5 cells/ml concentration (Supplementary Table S5).

### qPCRs

Bacteria and fungi were quantitatively analyzed using quantitative polymerase chain reaction (qPCR) duplex assays for parallel determination of Gram-positive and Gram-negative bacteria and total fungal DNA. The PCR protocol was followed as described in the original publication^44^, with minor modifications^45^. Microbial concentrations were presented as cell equivalents per milligram dust (CE/mg). Microbial loads—expressed as cell equivalents per square meter sampled floor area (CE/m^2^)—were calculated by multiplying microbial concentrations with the amounts of dust in the sample and dividing it by the sampling area (m^2^).

### Statistical analyses

Statistical analyses were performed using SAS (version 9.3; SAS Institute Inc., Cary, NC) and R software, and box-plots figures were drawn with RStudio version 3.6.1. Chi-squared test was used for categorical variables.

In LUKAS2, differences in alpha-diversity and relative abundance of individual genera in dust from the dog and non-dog homes were compared with Mann–Whitney U-test. Analysis of Composition of Microbiomes (ANCOM)^46^ was used to identify dog-associated taxa from phylum to genus level (ANCOM version 1.1-3 in R) only in LUKAS2, due to the low number of the dog homes in LISA and the unability of ANCOM or other similar methods to account for the stratified sampling used in LISA.

In LISA, sub samples were weighted in the analyses accordingly: random sample received a weight equal to the inverse of the proportion of individuals in the random sample (n = 250) out of the total individuals in the cohort (N = 1353) i.e. 1/(250/1353) = 5.412; and the asthma and ADHD enrichment samples received a weight equal to 1. Genus level taxa with significant results in LUKAS2 were replicated in LISA. In this replication, only taxa with assigned genus level taxonomy were included, i.e. taxa with genus “unknown” or “other” were excluded from analysis. The weighted data was analyzed using ‘sjstats’^47^ package in R software and differences between the dog and non-dog homes were calculated with weighted Mann–Whitney U-test.

In LUKAS2, phylogenetically informed bacterial composition differences in the dog homes and non-dog homes were analyzed with Adonis (R software, vegan package)^48^ by using bacterial weighted and unweighted Unifrac distance. Similarly was analyzed fungal data using Bray–Curtis and Binary Jaccard distance metrics. Adonis was adjusted for cat ownership, the number of older siblings, and whether there was snow on the ground when the sample was taken. No such statistical method was available for weighted data in LISA.

In addition, we analyzed the influence of dog ownership on the relative abundance of bacteria likely originating from human microbiota by utilizing a variable named “human source proxy” (HSP) that was created by summing up the relative abundances of two human microbiota associated genera (*Staphylococcus* and *Streptococcus*) and three families (*Corynebacteriaceae*, *Propionibacteriaceae*, and *Enterobacteriaceae*), as proposed earlier.

We also run the linear models between dog ownership and bacterial or fungal diversity (richness or Shannon index) and adjusted the models for cat ownership or/and indoor smoking (latter was possible only in LISA), the results between dog ownership and diversity remained (smoking results presented in Additional Table S6).

### Ethics approval and consent to participate

Written consents were acquired from the parents of the participating children. Ethical clearance has been applied for within the frameworks of the individual studies. All methods were carried out in accordance with relevant guidelines and regulations in the manuscript. Ethical approval for the LUKAS2 study has been provided by the research ethics committee of the Hospital District of Northern Savo, Kuopio, Finland (48/2004 LUKAS2) and for LISA study, the original study protocol was approved by the local ethics committee (the Bavarian Board of Physicians, reference number 01212).

## Supplementary information

Supplementary figure S1.

Supplementary figure S2.

Supplementary figure S3.

Supplementary figure S4.

Supplementary figure S5.

Supplementary figure S6.

Supplementary figure S7.

Supplementary figure S8.

Supplementary information.

## Data Availability

All sequencing data used in this study in LUKAS2 is available at the https://www.ebi.ac.uk/ena/data/view/PRJEB29081 website. In LISA cohort, due to data protection reasons, the datasets generated and/or analyzed during the current study cannot be made publicly available. The datasets are available to interested researchers from the corresponding author on reasonable request (e.g. reproducibility), provided the release is consistent with the consent given by the LISA study participants. Ethical approval might be obtained for the release and a data transfer agreement from the legal department of the Helmholtz Zentrum München must be accepted.

## Abbreviations

ANCOM: Analysis of composition of microbiomes
ADHD: Attention-deficit hyperactivity disorder
CE: Cell equivalent
FLASH: Fast length adjustment of short reads
HSP: Human source proxy
OTU: Operational taxonomic unit
PCoA: Principal coordinate analyses
qPCR: Quantitative polymerase chain reaction
QIIME: Quantitative insights into microbial ecology
