# Supplementary figures and images for: Associations between dog keeping and indoor dust microbiota
Source: Sci Rep. 2021 Mar 5;11:5341. doi: 10.1038/s41598-021-84790-w (PMC7935950; doi:10.1038/s41598-021-84790-w)

Shannon entropy

**A)**

$p=0.71$

$p=0.64$

$p=0.92$

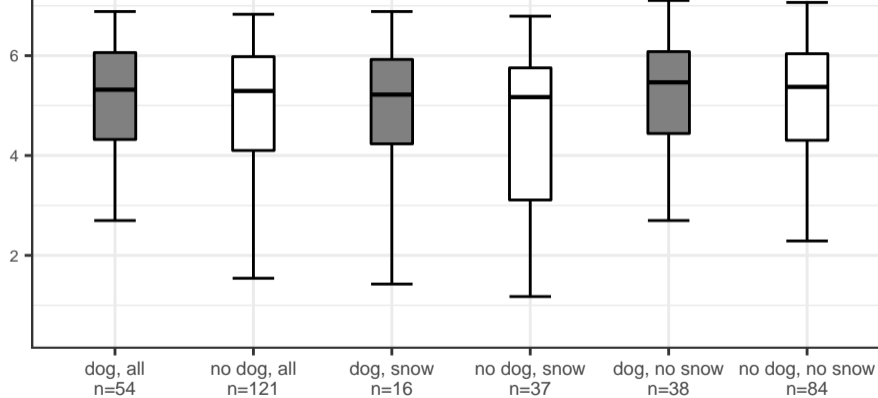

Dog ownership 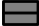 dog 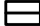 no dog

**B)**

$p=0.71$

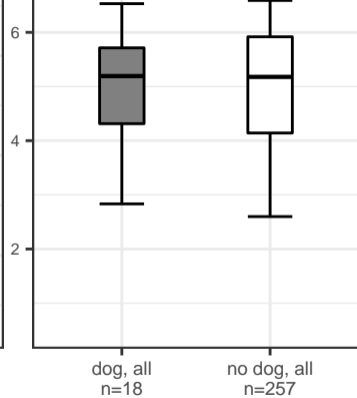

Supplement: Supplementary file 1 — Supplementary figure S1. [file 41598_2021_84790_MOESM1_ESM.pdf]

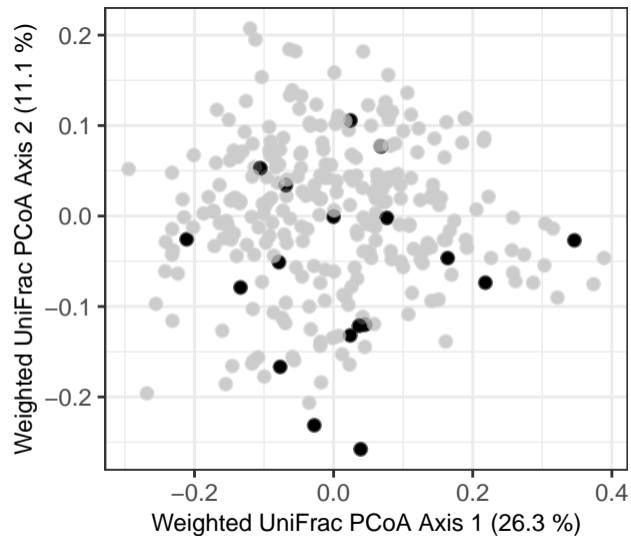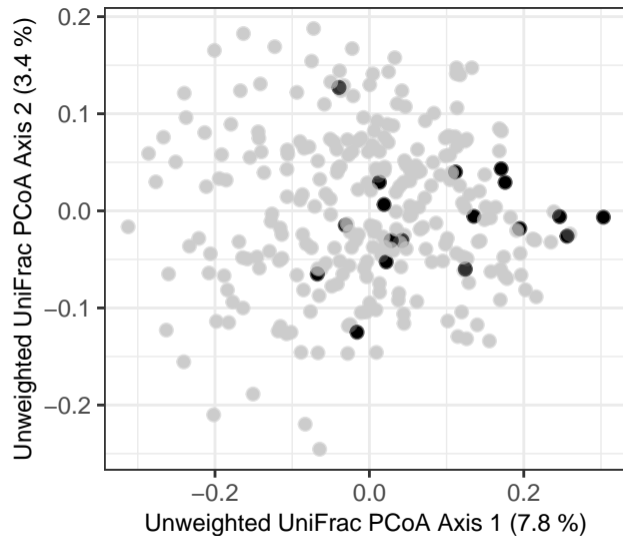

Dog ownership    ● No    ● Yes

Supplement: Supplementary file 2 — Supplementary figure S2. [file 41598_2021_84790_MOESM2_ESM.pdf]

## LUKAS2 cohort

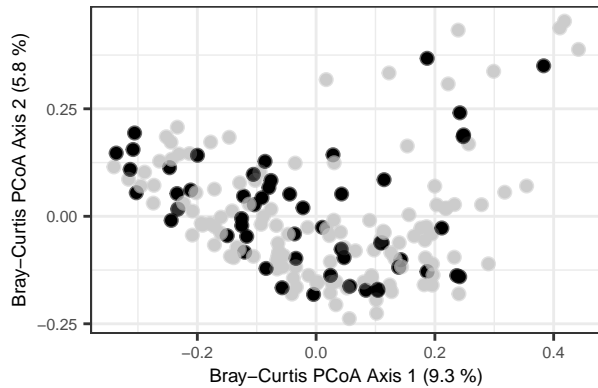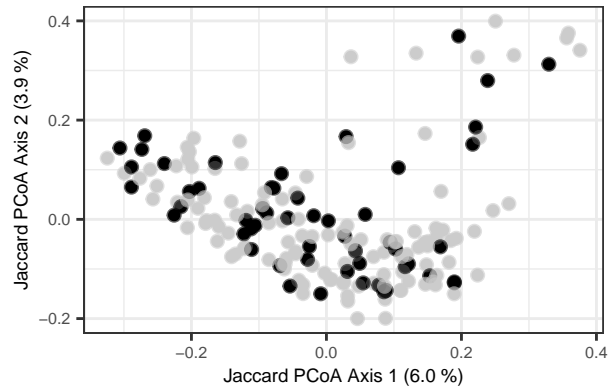

## During snow cover

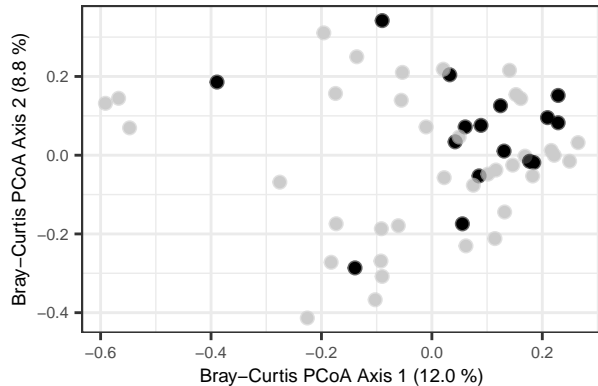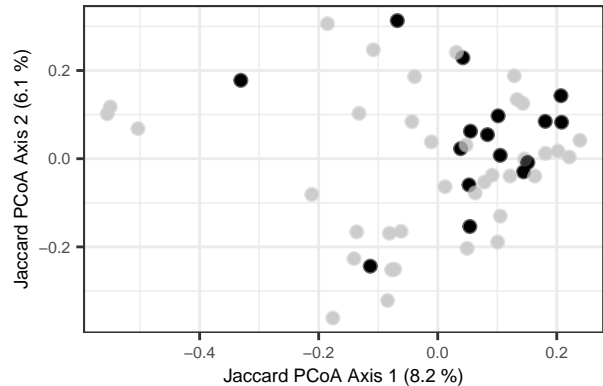

## Without snow cover

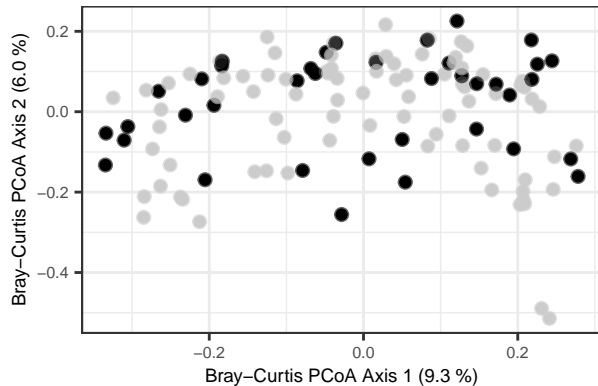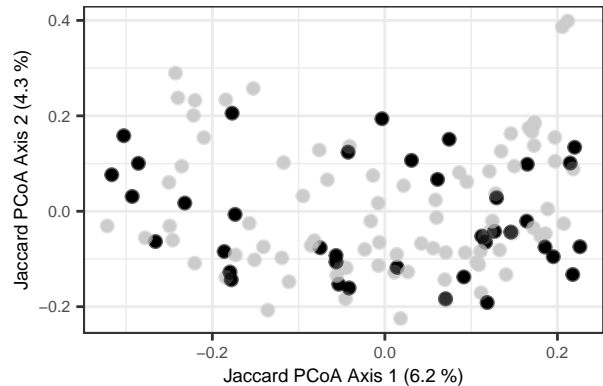

Dog ownership    ● No    ● Yes

Supplement: Supplementary file 3 — Supplementary figure S3. [file 41598_2021_84790_MOESM3_ESM.pdf]

Bray-Curtis PCoA Axis 2 (7.9 %)

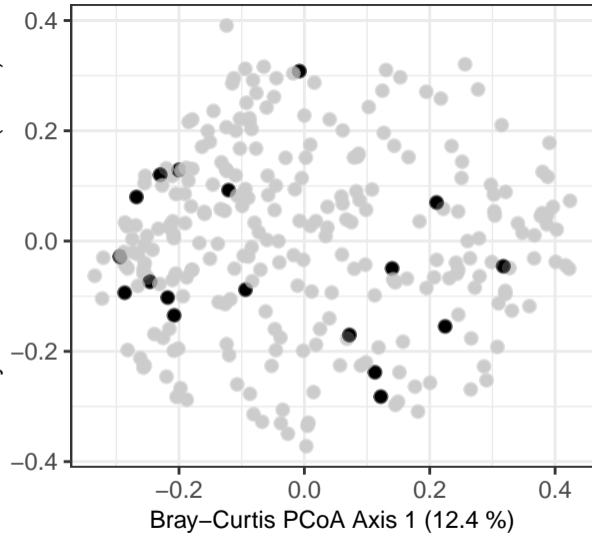

Jaccard PCoA Axis 2 (5.3 %)

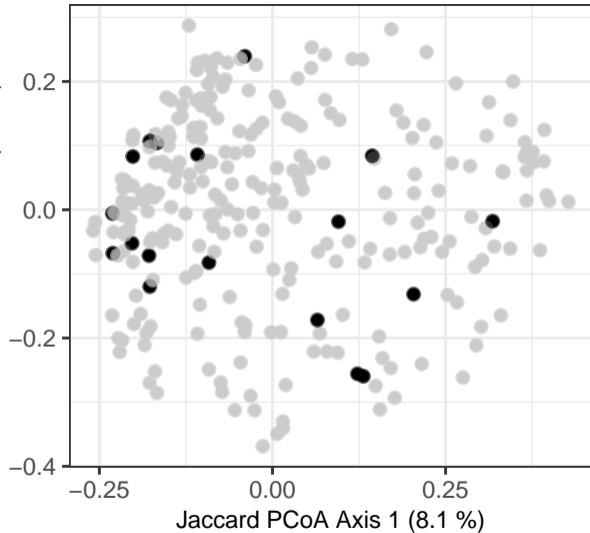

Dog ownership    ● No    ● Yes

Supplement: Supplementary file 4 — Supplementary figure S4. [file 41598_2021_84790_MOESM4_ESM.pdf]

## Total Bacteria

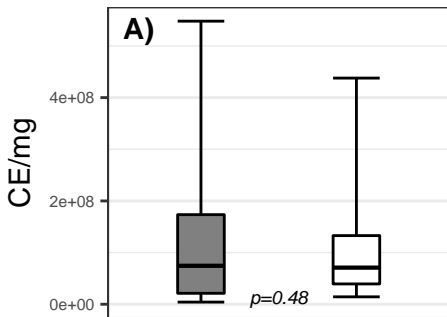

## Gram negative

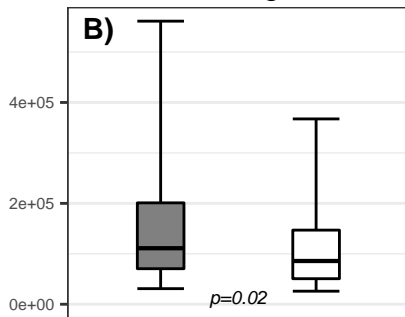

## Gram positive

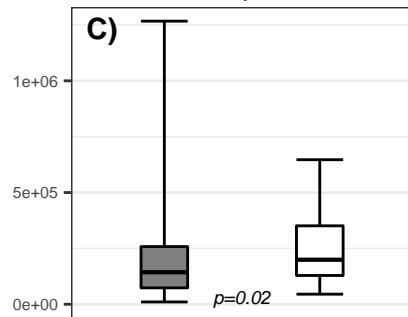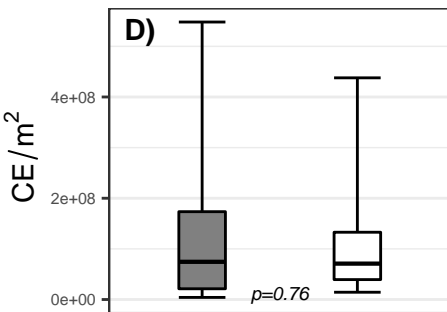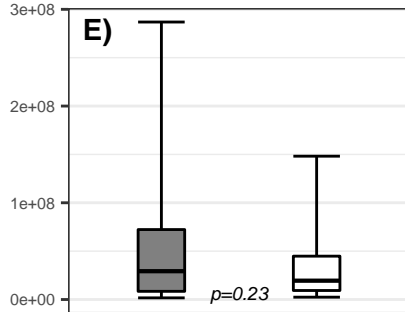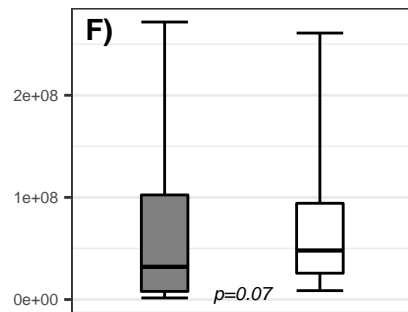

Dog ownership 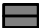 dog 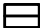 no dog

Supplement: Supplementary file 5 — Supplementary figure S5. [file 41598_2021_84790_MOESM5_ESM.pdf]

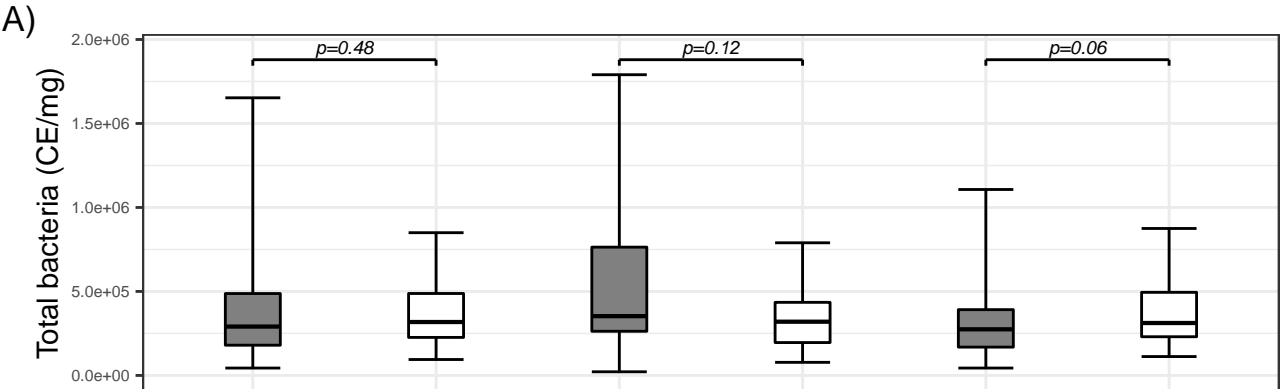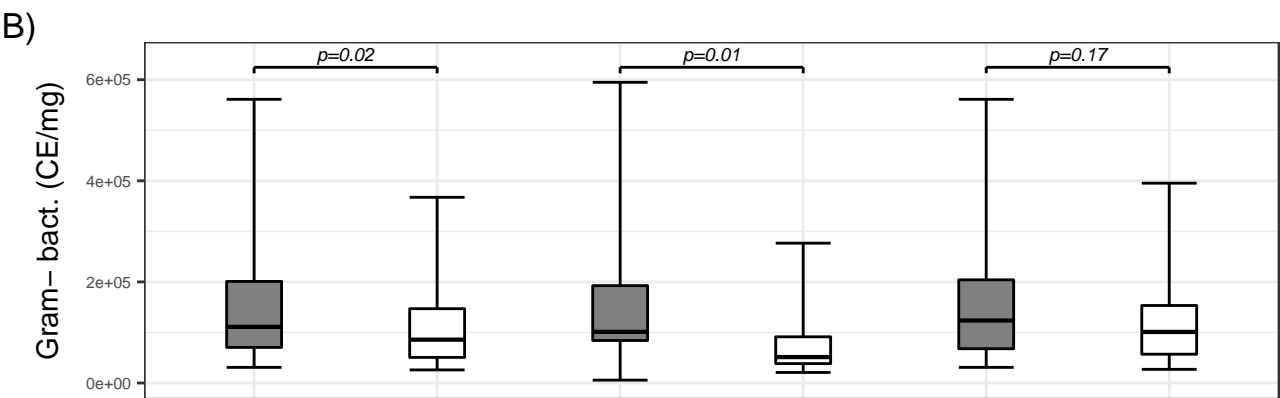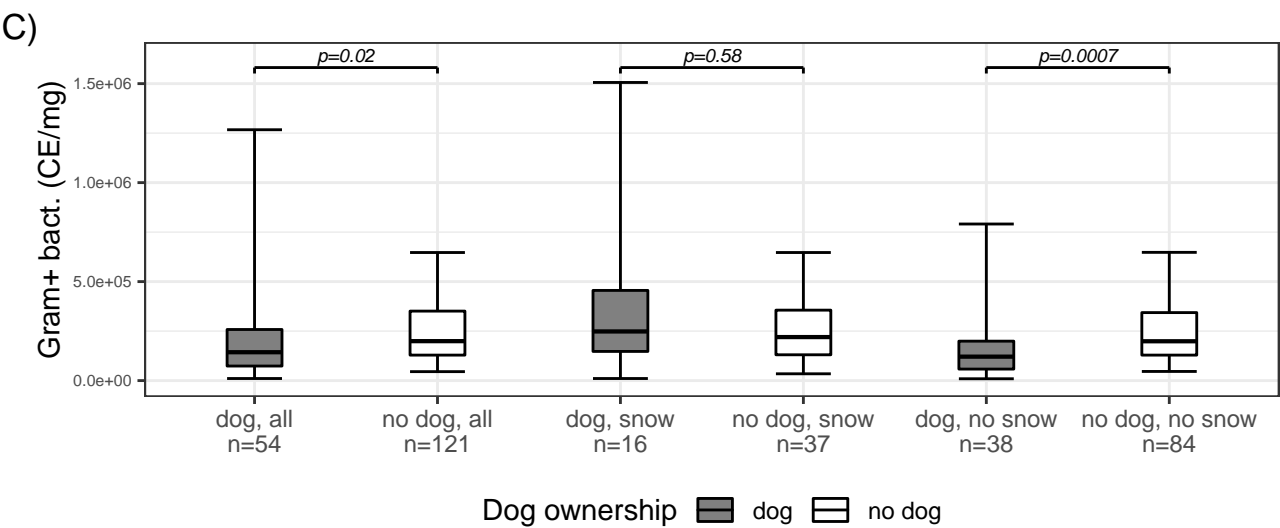

Supplement: Supplementary file 6 — Supplementary figure S6. [file 41598_2021_84790_MOESM6_ESM.pdf]

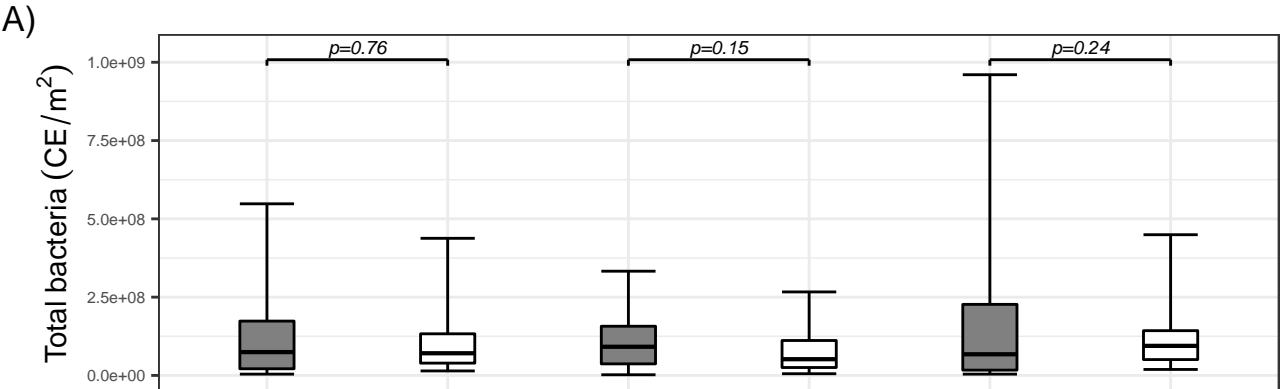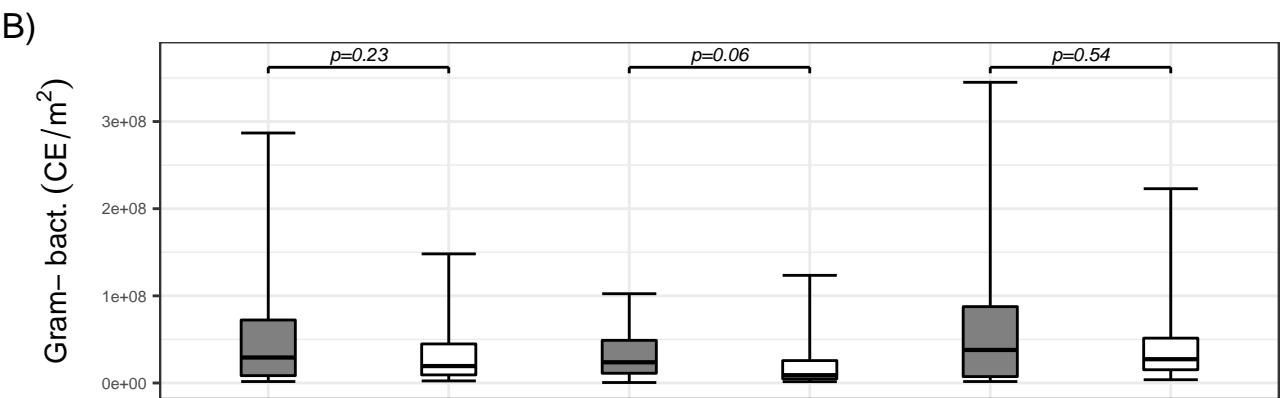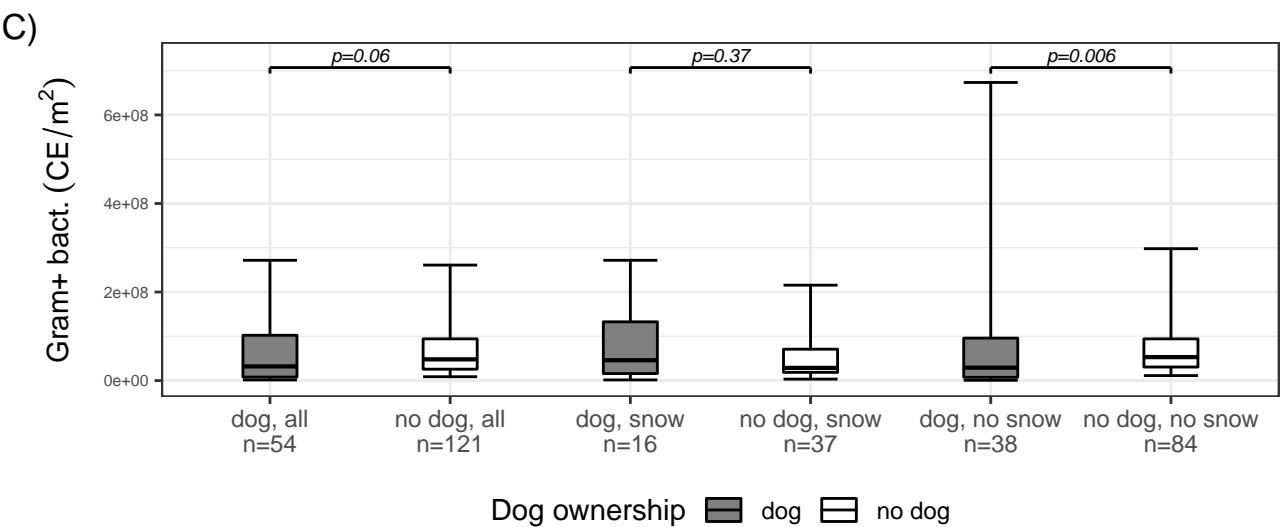

Supplement: Supplementary file 7 — Supplementary figure S7. [file 41598_2021_84790_MOESM7_ESM.pdf]

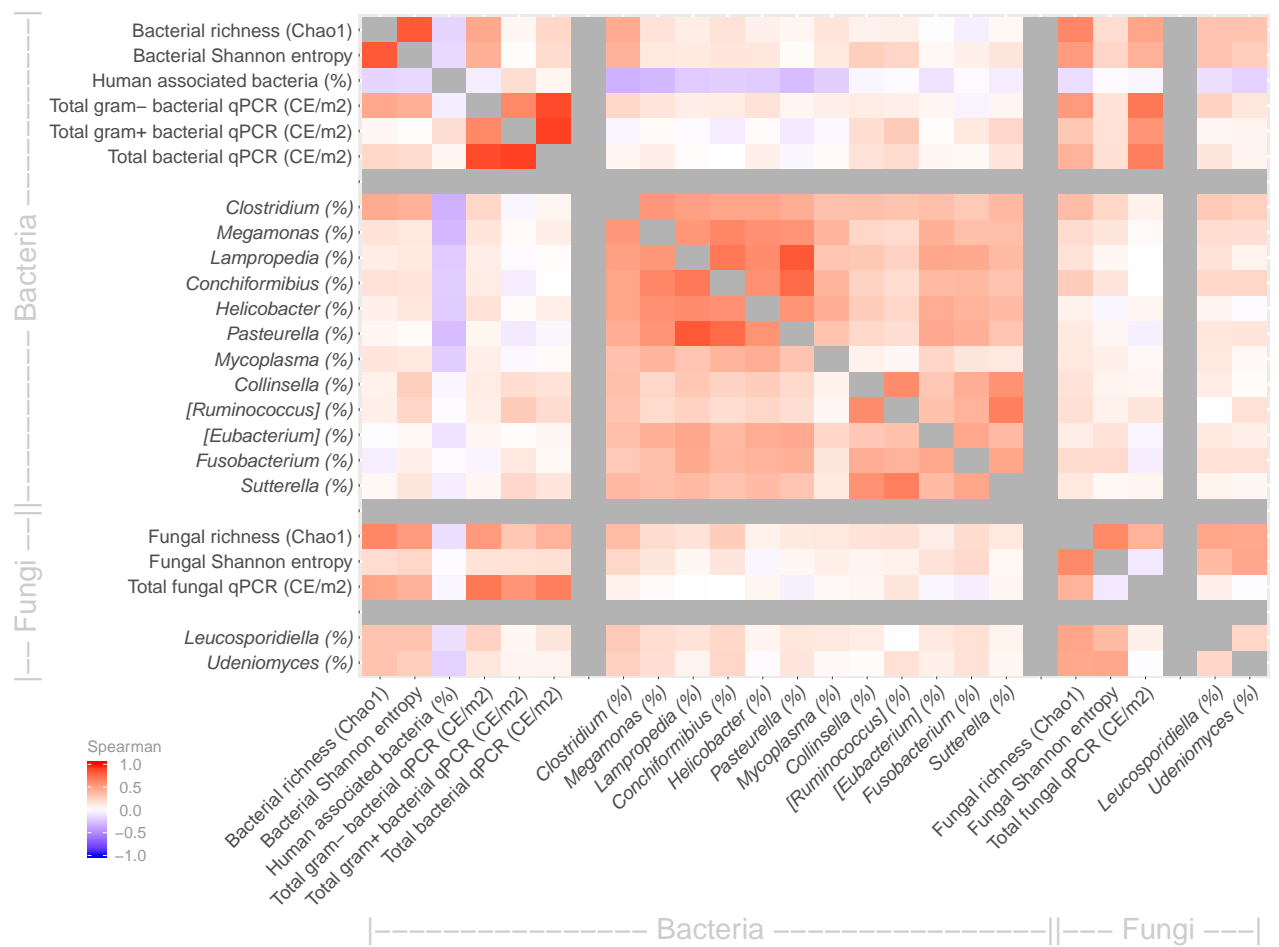

Supplement: Supplementary file 8 — Supplementary figure S8. [file 41598_2021_84790_MOESM8_ESM.pdf]
