# Supplementary material for: Associations between dog keeping and indoor dust microbiota
Source: Sci Rep. 2021 Mar 5;11:5341. doi: 10.1038/s41598-021-84790-w (PMC7935950; doi:10.1038/s41598-021-84790-w)
Supplement: Supplementary file 9 — Supplementary information. [file 41598_2021_84790_MOESM9_ESM.docx]

ADDITIONAL MATERIAL

**Associations between dog keeping and indoor dust microbiota**

Mäki JMK, Kirjavainen PV, Täubel M, Piippo-Savolainen E, Backman K, Hyvärinen A, Tuoresmäki P, Jayaprakash B, Heinrich J, Herberth G, Standl M, Pekkanen J, Karvonen AM

Contents

Methods………………………………………………………………………………………………………………………………………………….……2

Results………………………………………………………………………………………………………………………………………………………….4

References………………………………………………………………………………………………………………………………………….………..5

[Tables](#_Toc34060002) 6

Additional Table S1…………………………………………………………………………………………………………………………………..6

[Additional Table S2 7](#_Toc34060004)

[Additional Table S3 8](#_Toc34060005)

[Additional Table S4. 9](#_Toc34060006)

Additional Table S5……………………………………………………………………………………………………………………..………….10

Additional Table S6…………………………………………………………………………………………………………………………………11

[Figures 1](#_Toc34060007)2

[Additional Figure S1 1](#_Toc34060008)3

[Additional Figure S2 1](#_Toc34060009)4

[Additional Figure S3 1](#_Toc34060010)5

[Additional Figure S4 1](#_Toc34060011)6

[Additional Figure S5 1](#_Toc34060012)7

Additional Figure S6……………………………………………………………………………………………………………………………….18

[Additional Figure S7 1](#_Toc34060013)9

[Additional Figure S8](#_Toc34060014) 20

**Methods**

*DNA extraction, 16S rRNA gene and ITS PCR and amplicon sequencing*

DNA extraction was carried out from a target amount of 20 mg of dust including a bead milling step and clean-up with Chemagic DNA Plant–kit (PerkinElmer chemagen Technologie GmbH, Germany) on a KingFisher DNA extraction robot. DNA was shipped frozen to a commercial sequencing service partner (LGC Genomics, Germany) that carried out PCR amplification and sequencing of these amplicons. The bacterial 16S rRNA gene V4 region was amplified using 515F/806R primers [1] and fungal ITS region by ITS1F/ITS2 primers [2] and amplicons were sequenced with Illumina MiSeq V3 Chemistry (300 base pair paired end reads). Post de-multiplexing, barcode sorting/clipping, adapter and primer clipping, the forward and reverse reads were merged using FLASH (Fast Length Adjustment of SHort reads) software [3]. Subsequent processing and analysis steps including initial quality checks and chimera removal relied largely on QIIME (Quantitative Insights Into Microbial Ecology); [4] software version 1.9.1, complemented by additional software. Details of the sequence processing are presented in the earlier publications that have first described sequence processing in the LISA [5] and LUKAS cohorts [6, 7]. Sequences were clustered into operational taxonomic units (OTUs) at 97% similarity using the open-reference approach with the greengenes 16S rRNA gene database for bacteria, and the ITS database UNITE for fungi [8, 9]. Alpha diversity indices Chao1 and Shannon were calculated with QIIME. In LUKAS2, rarefaction was done at 2150 sequences for bacteria and 2700 sequences for fungi. In LISA, the rarefaction values were 1020 and 990 sequences for bacteria and fungi, respectively. For the analysis of individual fungal taxa, taxonomic classification was done using FHiTINGS (Fungal High throughput Taxonomy Identification in NGS), calculating taxa-based OTU groups instead of clustering [10]. Negative (reagents) and positive (bacterial and fungal mock community) controls were included in the DNA extraction alongside processing of the actual house dust samples. The bacterial mock community consisted of equal concentration 1E+8 cells/ml of seven species, while the fungal mock community was an assemblage of 43 species at either 1E+4 or 1E+5 cells/ml concentration (Supplementary Table 5). Negative reagent controls were included in the sequence processing of the samples in order to identify contaminants and to inform sequence number threshold for inclusion of samples in downstream analyses and rarefaction in alpha and beta diversity calculations.

For bacteria, the mock community consisting of seven known taxa was completely recovered on genus or family level in both LUKAS and LISA studies, with the exception of *Pseudomonas aeruginosa* that was missing from the community profile of the bacterial mocks. For fungi, 43 taxa were in the reference mock community and all but seven taxa (*Acremonium strictum, Eurotium chevalieri, Mucor racemosus, Rhizopus stolonifera, Stachybotrys chartarum, Ulocladium* spp., *Wallemia sebi*) had OTUs consistently - across fungal mocks in the two datasets - represented by taxa of the genus, however not necessarily to the precise species.

*qPCRs*

Bacteria and fungi were quantitatively analyzed using quantitative polymerase chain reaction (qPCR) duplex assays for parallel determination of Gram-positive and Gram-negative bacteria and total fungal DNA. The PCR protocol was followed as described in the original publication [11], with minor modifications [12]. Salmon testis DNA was used as an internal standard to allow correcting errors resulting from amplification inhibition and DNA loss during extraction and clean-up [13]. Equivalents of microbial cell numbers in the samples were calculated using relative quantification [14]. Microbial concentrations were presented as cell equivalents per milligram dust (CE/mg). Microbial loads - expressed as cell equivalents per square meter sampled floor area (CE/m^2^) - were calculated by multiplying microbial concentrations with the amounts of dust in the sample and dividing it by the sampling area (m^2^).

**Results**

*LISA subgroup analyses*

In LISA, 120 families had dust samples available from bedroom and living room. Within these samples, the bacterial richness and Shannon entropy were only moderately correlated (Spearman correlation, *r=0.39 and 0.36*, respectively) between two locations while somewhat stronger correlations were seen with the fungal diversity indices (*r=0.55 and 0.58*, respectively). The means of bacterial diversity indices and fungal richness were higher in the living room samples than in the bedroom samples (mean 819.8 *vs.* 766.2, paired T-test *p=0.004* for bacterial richness; 6.6 *vs.* 6.4, *p=0.02* for bacterial Shannon entropy; and 301.6 *vs.* 286.7, *p=0.04* for fungal richness, respectively), however, the mean of fungal Shannon entropy was a bit lower in living rooms than in bedrooms (4.9 *vs.* 5.1, *p=0.06*). HSP correlated moderately (*r=0.43*) and the levels were significantly higher in bedrooms than in living rooms (mean 0.42 *vs.* 0.37, *p=0.0005*).

References

1. Caporaso JG, *et al* Global patterns of 16S rRNA diversity at a depth of millions of sequences per sample. *Proc Natl Acad Sci U S A* , **108** Suppl 1:4516-4522 (2011)

2. Smith DP, Peay KG: Sequence depth, not PCR replication, improves ecological inference from next generation DNA sequencing. *PLoS One* **9** e90234 doi:10.1371/journal.pone.0090234 (2014)

3. Magoc T, Salzberg SL: FLASH: fast length adjustment of short reads to improve genome assemblies. *Bioinformatics* **27,** 2957-2963 (2011)

4. Caporaso, J.G. *et al.* QIIME allows analysis of high-throughput community sequencing data. *Nat. Methods* **7**, 335-336 (2010).

5. Casas, L. *et al.* Early life home microbiome and hyperactivity/inattention in school-age children. *Scientific Reports* **9**, 17355; 10.1038/s41598-019-53527-1 (2019).

6. Kirjavainen, P.V. *et al.* Farm-like indoor microbiota in non-farm homes protects children from asthma development. *Nat. Med.* **25**, 1089-1095 (2019).

7. Karvonen AM*, et al*: Indoor bacterial microbiota and development of asthma by 10.5 years of age. *Journal of Allergy and Clinical Immunology* **144,** 1402-10 (2019).

8. DeSantis T.Z, *et al*. Greengenes, a Chimera-Checked 16S rRNA Gene Database and Workbench Compatible with ARB. *Applied and Environmental Microbiology* **72**, 5069-5072 (2006).

9. Abarenkov K. *et al*. The UNITE database for molecular identification of fungi – recent updates and future perspectives. *The New Phytologist* **186**, 281-285 (2010).

10. Dannemiller KC, Reeves D, Bibby K, Yamamoto N, Peccia J: Fungal High‐throughput Taxonomic Identification tool for use with Next‐Generation Sequencing (FHiTINGS). *Journal of Basic Microbiology*  **54**, 315-321 (2014).

11. Karkkainen PM, Valkonen M, Hyvarinen A, Nevalainen A, Rintala H: Determination of bacterial load in house dust using qPCR, chemical markers and culture. *J Environ Monit* **12**, 759-768 (2010).

12. Jayaprakash B, *et al*. Indoor microbiota in severely moisture damaged homes and the impact of interventions. *Microbiome* **5**, 138-5 (2017).

13. Haugland RA, Siefring SC, Wymer LJ, Brenner KP, Dufour AP: Comparison of Enterococcus measurements in freshwater at two recreational beaches by quantitative polymerase chain reaction and membrane filter culture analysis. *Water Research* **39**, 559-568 (2005)

14. Haugland RA, Varma M, Wymer LJ, Vesper SJ: Quantitative PCR Analysis of Selected Aspergillus, Penicillium and Paecilomyces Species. *Systematic and Applied Microbiology* **27**, 198-210 (2004)

# Tables

Additional Table S1. Characteristics of the study populations and comparisons between households with and without dogs.

N the number of observations, n the number of observations in the given group, % the percentage of the observations within dog households and without dog. P-values are from chi square test. -- cannot be estimated.

Additional Table S2. Bacterial taxa with different levels of relative abundance in households with dogs than without dogs in LUKAS2.


 Bacterial taxa with significantly (two-sided corrected P-value <0.05) different relative abundance in the living room floor dust of households with dog (n=56) than without dogs (n=126) among LUKAS2 non-farmers as defined by Ancom. Taxa in the table are sorted by taxonomic level. D* Direction of the comparison: ↑ more abundant in households with dogs than without dogs; ↓ less abundant in households with dogs than without dogs. Unassigned= sum of sequences with non-resolved (unassigned) taxonomy at the respective taxon level; Other= sum of sequences not assigned to database entries at the respective taxon level.

**Additional** Table S3. Comparisons between relative abundances of bacterial and fungal genera in households with and without dog.

Table shows genera which were identified exclusively from dust samples while snow covered ground. In LUKAS2, results are presented separately for samples collected when snow covered the ground and without snow cover. The number of dog homes (the number of no dog homes) in bacterial and fungal data in LUKAS2 during snow cover: 17 (37) and 16 (37) The number of dog homes (the number of no dog homes) in bacterial and fungal data in LUKAS2 collected without snow cover: 39 (89) and 38 (84) The number of dog homes (the number of no dog homes) in bacterial and fungal data in LISA: 18 (262) and 18 (257) p- values are from Mann-Whitney U –test in LUKAS2 and weighted Mann-Whitney U –test in LISA.

**Additional** Table S4. Fungal taxa with different levels of relative abundance in households with dogs than without dogs in LUKAS2.

Fungal taxa with significantly (two-sided corrected P-value <0.05) higher or lower relative abundance in the living room floor dust of dog homes (n=54) than non-dog homes (n=121) among LUKAS2 non-farmers as defined by Ancom. Taxa in the table are sorted by taxonomic level. D* Direction of the comparison: ↑ more abundant in households with dogs than without dogs; ↓ less abundant in households with dogs than without dogs. incertae sedis is used for a taxon when its broader relationship to other taxa is unknown, i.e. its positing in the current taxonomy is not resolved.

# Table S5. Bacterial and fungal mock communities.

| **Bacterial mock community** |  |  | **Fungal mock community** |  |  |
| --- | --- | --- | --- | --- | --- |
| Species | cells/mL |  | Species | cells/mL |  |
| Staphylococcus aureus | 1.00E+08 |  | Acremonium strictum | 1.00E+05 |  |
| Streptomyces californicus | 1.00E+08 |  | Alternaria alternata | 1.00E+04 |  |
| Bacillus cereus | 1.00E+08 |  | Aspergillus flavus | 1.00E+05 |  |
| E. coli | 1.00E+08 |  | Aspergillus fumigatus | 1.00E+05 |  |
| Pseudomonas aeruginosa | 1.00E+08 |  | Aspergillus niger | 1.00E+05 |  |
| Sphingomonas faeni | 1.00E+08 |  | Aspergillus ochraceus | 1.00E+05 |  |
| Mycobacteriun mucogenicum | 1.00E+08 |  | Aspergillus penicillioides | 1.00E+05 |  |
|  |  |  | Aspergillus restrictus | 1.00E+05 |  |
|  |  |  | Aspergillus sclerotiorum | 1.00E+05 |  |
|  |  |  | Aspergillus sydowii | 1.00E+05 |  |
|  |  |  | Aspergillus terreus | 1.00E+05 |  |
|  |  |  | Aspergillus unguis | 1.00E+05 |  |
|  |  |  | Aspergillus ustus | 1.00E+05 |  |
|  |  |  | Aspergillus versicolor | 1.00E+05 |  |
|  |  |  | Chaetomium globosum | 1.00E+05 |  |
|  |  |  | Cladosporium cladosporioides- Type 1 | 1.00E+05 |  |
|  |  |  | Cladosporium cladosporioides- Type 2 | 1.00E+05 |  |
|  |  |  | Cladosporium herbarum | 1.00E+05 |  |
|  |  |  | Cladosporium sphaerospermum | 1.00E+05 |  |
|  |  |  | Eurotium chevalieri | 1.00E+05 |  |
|  |  |  | Mucor racemosus | 1.00E+05 |  |
|  |  |  | Paecilomyces varioti | 1.00E+05 |  |
|  |  |  | Penicillium brevicompactum | 1.00E+05 |  |
|  |  |  | Penicillium chrysogenum type 2 | 1.00E+05 |  |
|  |  |  | Penicillium citrinum | 1.00E+05 |  |
|  |  |  | Penicillium corylophilum | 1.00E+05 |  |
|  |  |  | Penicillium expansum | 1.00E+05 |  |
|  |  |  | Penicillium italicum | 1.00E+05 |  |
|  |  |  | Penicillium oxalicum | 1.00E+05 |  |
|  |  |  | Penicillium purpurogenum | 1.00E+05 |  |
|  |  |  | Penicillium sclerotiorum | 1.00E+05 |  |
|  |  |  | Penicillium spinulosum | 1.00E+05 |  |
|  |  |  | Penicillium variabile | 1.00E+05 |  |
|  |  |  | Rhizopus stolonifer | 1.00E+05 |  |
|  |  |  | Scopulariopsis brevicaulis | 1.00E+05 |  |
|  |  |  | Scopulariopsis chartarum | 1.00E+05 |  |
|  |  |  | Stachybotrys chartarum | 1.00E+04 |  |
|  |  |  | Trichoderma harzianum | 1.00E+05 |  |
|  |  |  | Trichoderma longibrachiatum | 1.00E+05 |  |
|  |  |  | Trichoderma viride type 1 | 1.00E+05 |  |
|  |  |  | Ulocladium chartarum | 1.00E+04 |  |
|  |  |  | Ulocladium botrytis | 1.00E+04 |  |
|  |  |  | Wallemia sebi | 1.00E+05 |  |

**Additional Table S6.** Associations between dog ownership and bacterial and fungal diversities in LISA cohort, additionally adjusted for passive smoking.

|  | Model 1 | |  | Model 2 | |
| --- | --- | --- | --- | --- | --- |
|  | F | *p* |  | F | *p* |
| **Bacteria** |  |  |  |  |  |
| **Chao1** |  |  |  |  |  |
| Dog | 11.04 | *0.001* |  | 11.29 | *0.0009* |
| Passive smoking | - | *-* |  | 1.07 | *0.30* |
| **Shannon entropy** |  |  |  |  |  |
| Dog | 8.29 | *0.004* |  | 8.27 | *0.003* |
| Passive smoking | - | *-* |  | 0.99 | *0.32* |
| **Fungi** |  |  |  |  |  |
| **Chao1** |  |  |  |  |  |
| Dog | 0.14 | *0.71* |  | 0.07 | *0.79* |
| Passive smoking | - | *-* |  | 1.82 | *0.18* |
| **Shannon entropy** |  |  |  |  |  |
| Dog | 0.55 | *0.46* |  | 0.51 | *0.48* |
| Passive smoking | - | *-* |  | 0.17 | *0.68* |

N Number of observations in the model: in bacterial and fungal model 1: 280 and 275; and in model 2: 277 and 272, respectively. F and *p*-values are from the weighted generalized linear models. Model 1: dog vs. diversity; Model 2: Model 1 is adjusted for passive smoking.

**Figures**


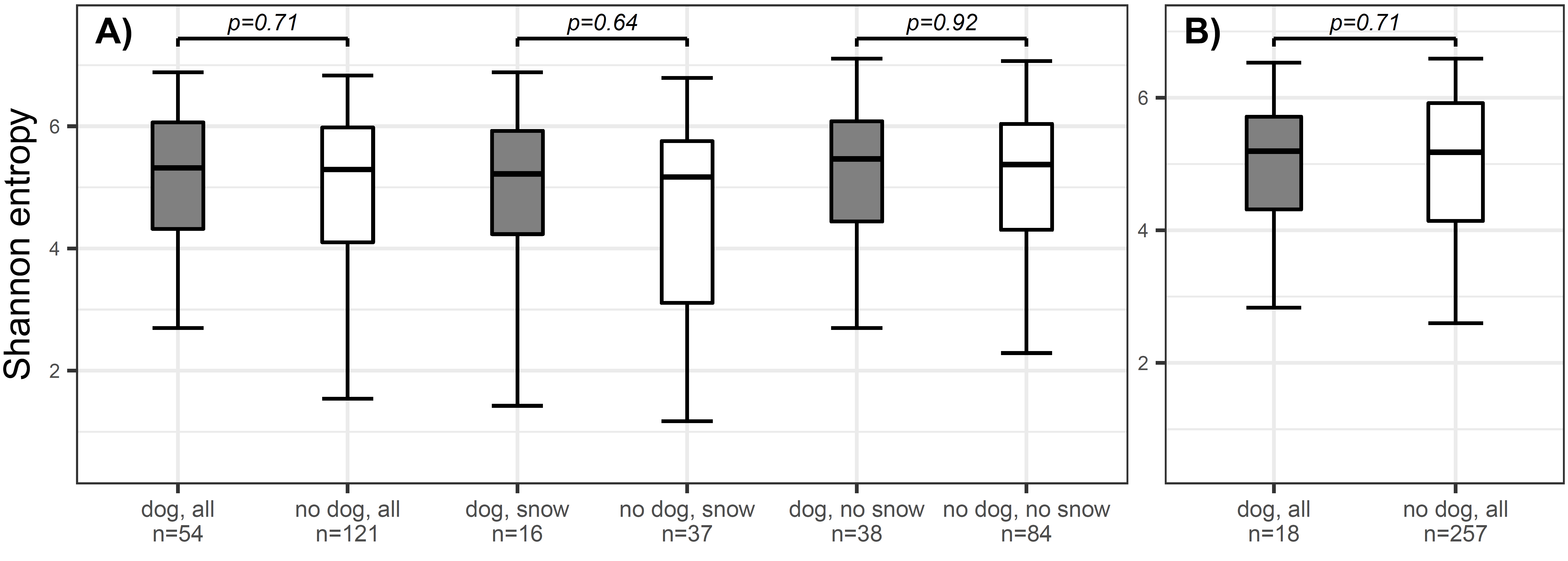


**Additional** Figure S1. **):** Box-plots of fungal Shannon entropy in dust from the dog and non-dog homes in two cohorts. Dust samples collected from dog homes (grey) and non-dog homes (white) in A) LUKAS2 and B) LISA cohorts. In LUKAS2, additional comparison is made by samples taken during snow cover (snow) and without snow cover (no snow). *P*-values are from Mann-Whitney U –test in LUKAS2 and weighted Mann-Whitney U –test in LISA. The boxplots present 5^th^ percentile, first quartile, median, third quartile, and 95^th^ percentile of the values.


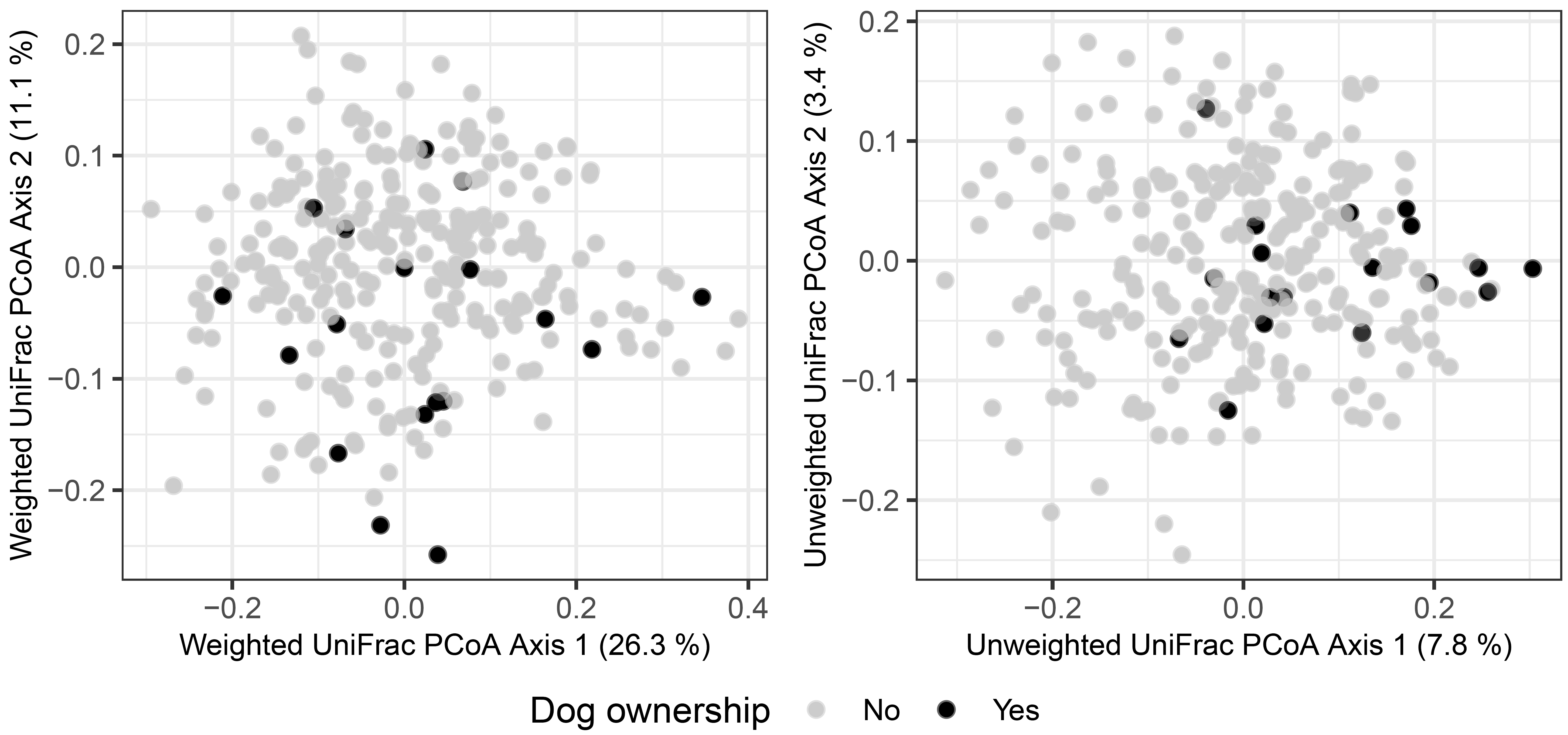


Additional Figure S2**.** Plots of PCoA1 and PCoA2 two bacterial axes scores by dog houses in LISA cohort. Plots of the first (PCoA1) and the second (PCoA2) axes scores from bacterial weighted and unweighted UniFrac based Principal Coordinate Analyses in LISA cohort by houses with (black dots) or without (grey dots) dog(s). Percentage of the variance explained by the axis score is in the parentheses. Plot of PCoA1 and PCoA2 axes scores by dog houses in bacterial weighted and unweighted UniFrac distances in LISA cohort. PCoA1 is the first and PCoA2 the second axis scores from weighted or unweighted UniFrac based Principal Coordinate Analyses; dog homes (black dots) and non-dog homes (grey dots); percentages of the variance explained by the axis scores are in the parentheses.


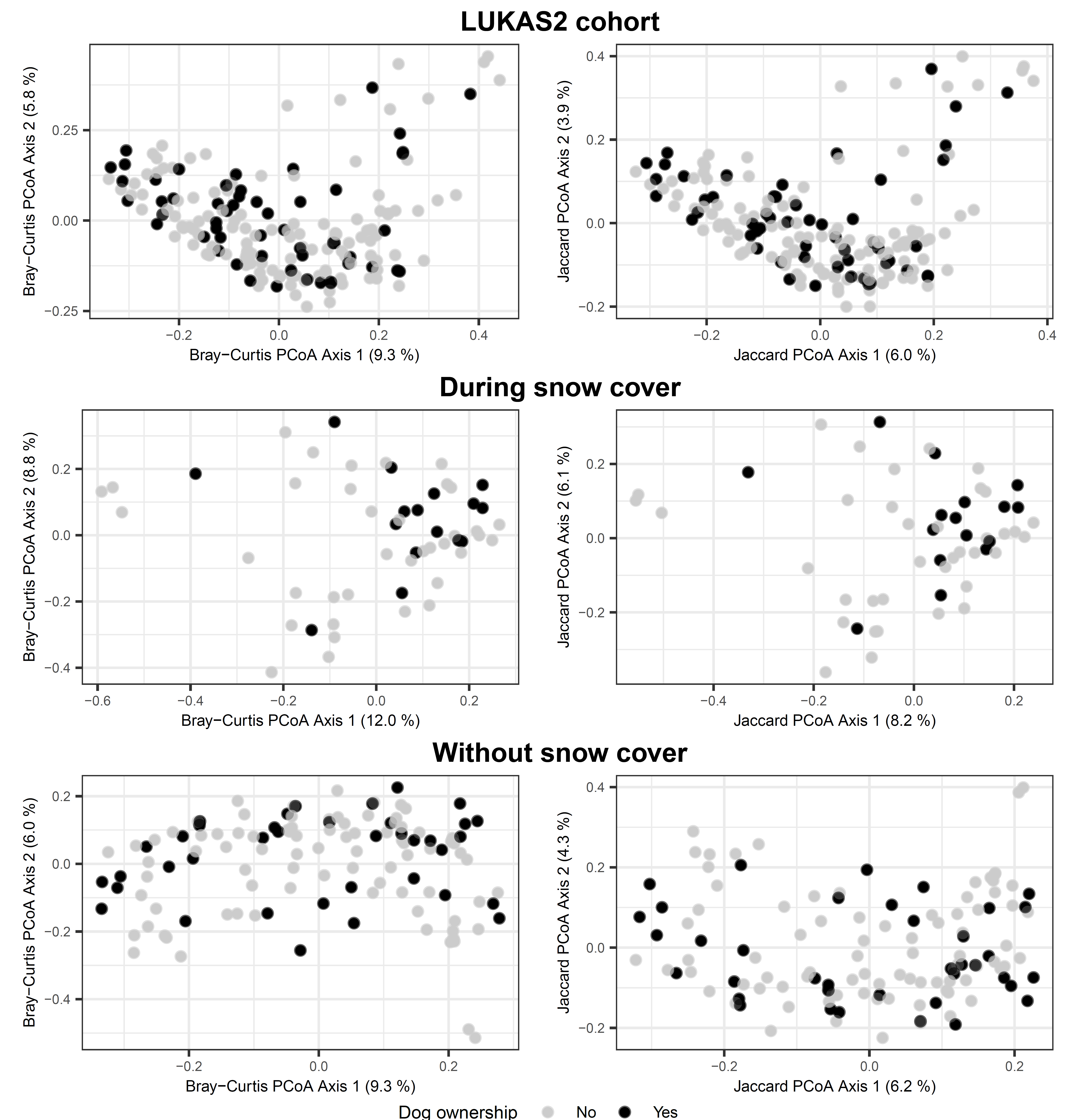


Additional Figure S3**.** Plots of two fungal axes scores by dog houses stratified by snow cover in LUKAS2. Plots of the first (PCoA1) and the second (PCoA2) axes scores from fungal Bray Curtis and Jaccard based Principal Coordinate Analyses in LUKAS2 cohort in house dust samples collected from homes with (black dots) or without (grey dots) dog(s). Percentage of the variance explained by the axis score is in the parentheses. Top two plots were made using dust samples from the whole LUKAS2 cohort (excl. farmers), two middle plots with samples collected without snow cover, and two plots in the bottom with samples when snow was on the ground.


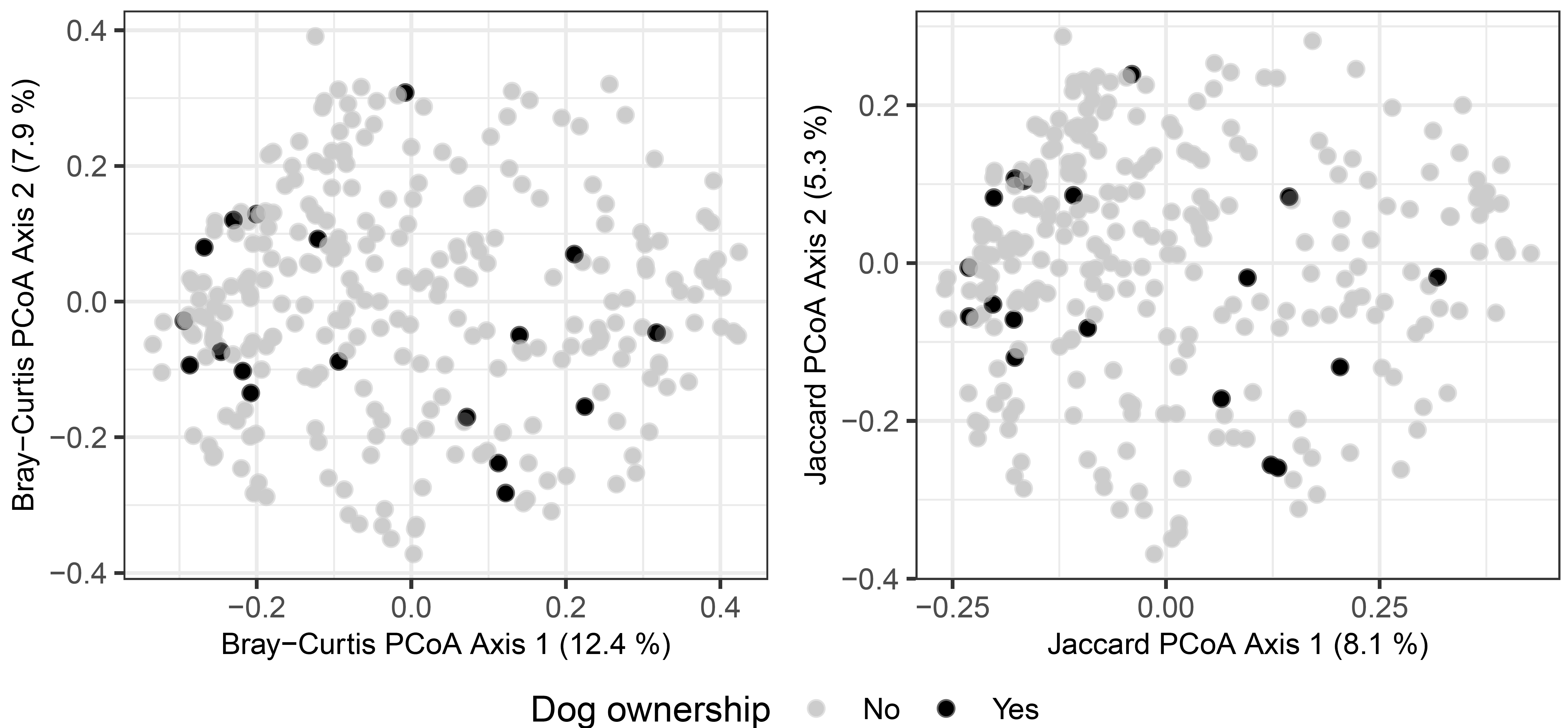


**Additional** Figure S4. Plots of two fungal axes scores by dog houses in LISA cohort. Plots of the first (PCoA1) and the second (PCoA2) axes scores from fungal Bray Curtis and Jaccard based Principal Coordinate Analyses in LISA cohort by houses with (black dots) or without (grey dots) dog(s). Percentage of the variance explained by the axis score is in the parentheses.


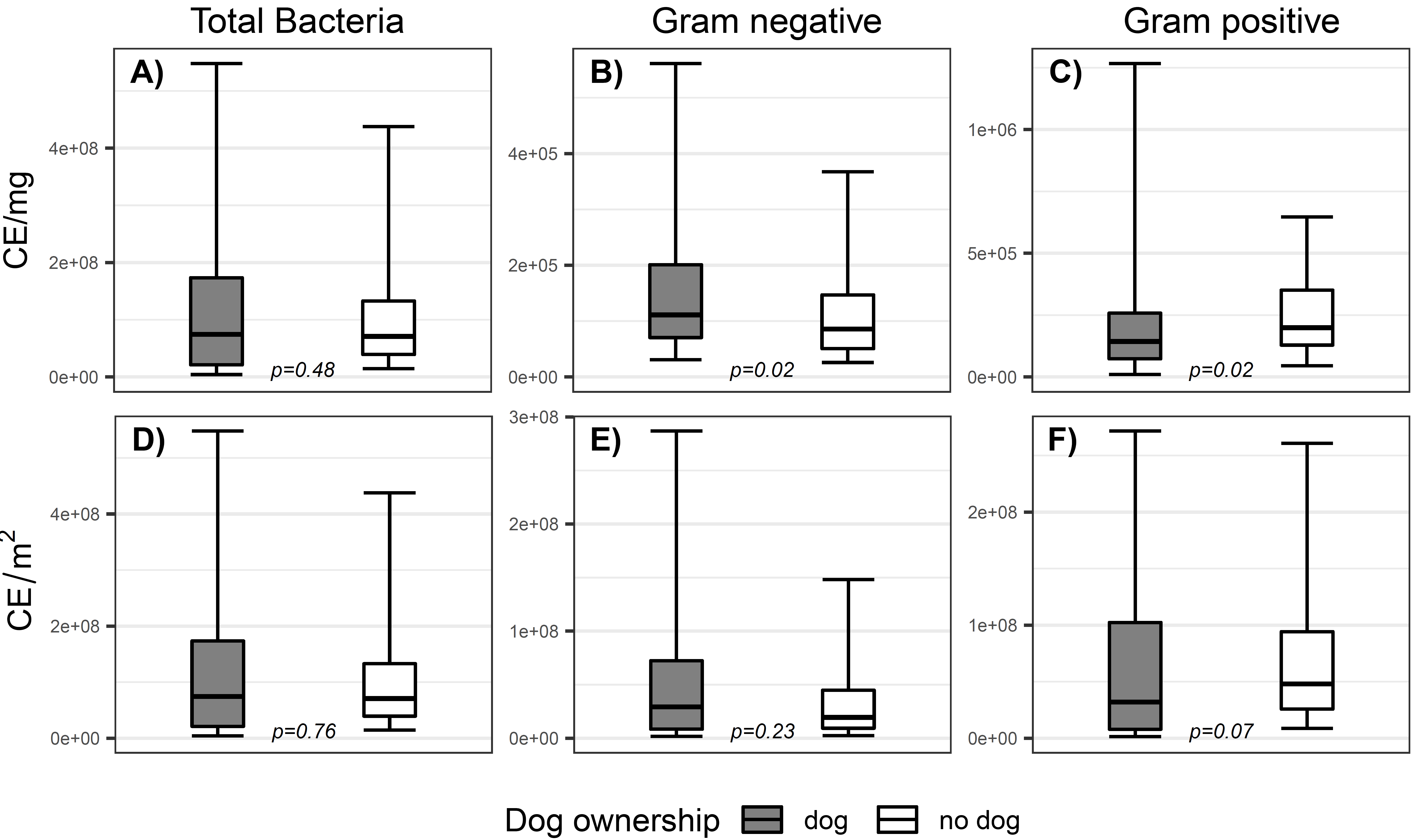


**Additional** Figure S5. Box-plots of total, Gram-negative and Gram-positive bacteria in house dust in LUKAS2. Box-plots of the concentrations (cell equivalent [CE] per mg of dust) A) total bacteria, B) Gram-negative (Gram- bact.) and C) Gram-positive (Gram+ bact.) bacteria, and of the loads (CE per square meter of floor area) of D) total bacteria, E) Gram-negative and F) Gram-positive bacteria in house dust samples collected from dog homes (grey) and non-dog homes (white) in the whole LUKAS2 (excl. farmers). P-values are from Mann-Whitney U-test. The boxplots present 5^th^ percentile, first quartile, median, third quartile, and 95^th^ percentile of the values.


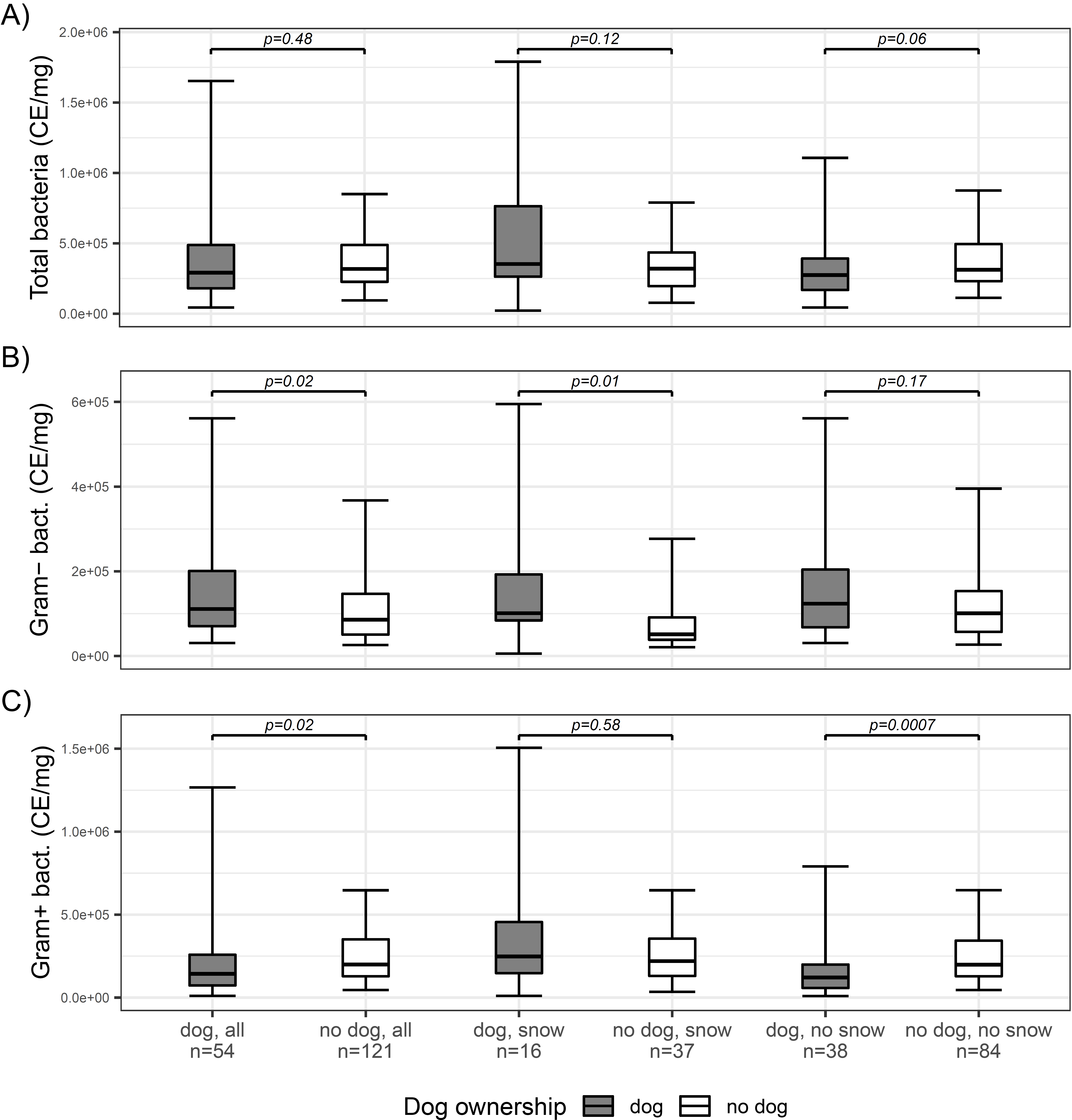


**Additional** Figure **S6.** Box-plots of the bacterial qPCR results (concentrations) in LUKAS2, stratified by snow cover. Box-plots of the levels of concentrations (cell equivalent [CE] per mg of dust) of quantitative polymerase chain reaction (qPCR): A) total bacteria, B) Gram-negative (Gram- bact.) and C) Gram-positive (Gram+ bact.) bacteria in house dust samples collected from dog homes (grey) and non-dog homes (white) in LUKAS2 (all), and stratified by snow cover (snow=dust samples collected when snow covered the ground; no snow=collected without snow cover). P-values from Mann-Whitney U-test. The boxplots present 5^th^ percentile, first quartile, median, third quartile, and 95^th^ percentile of the values.


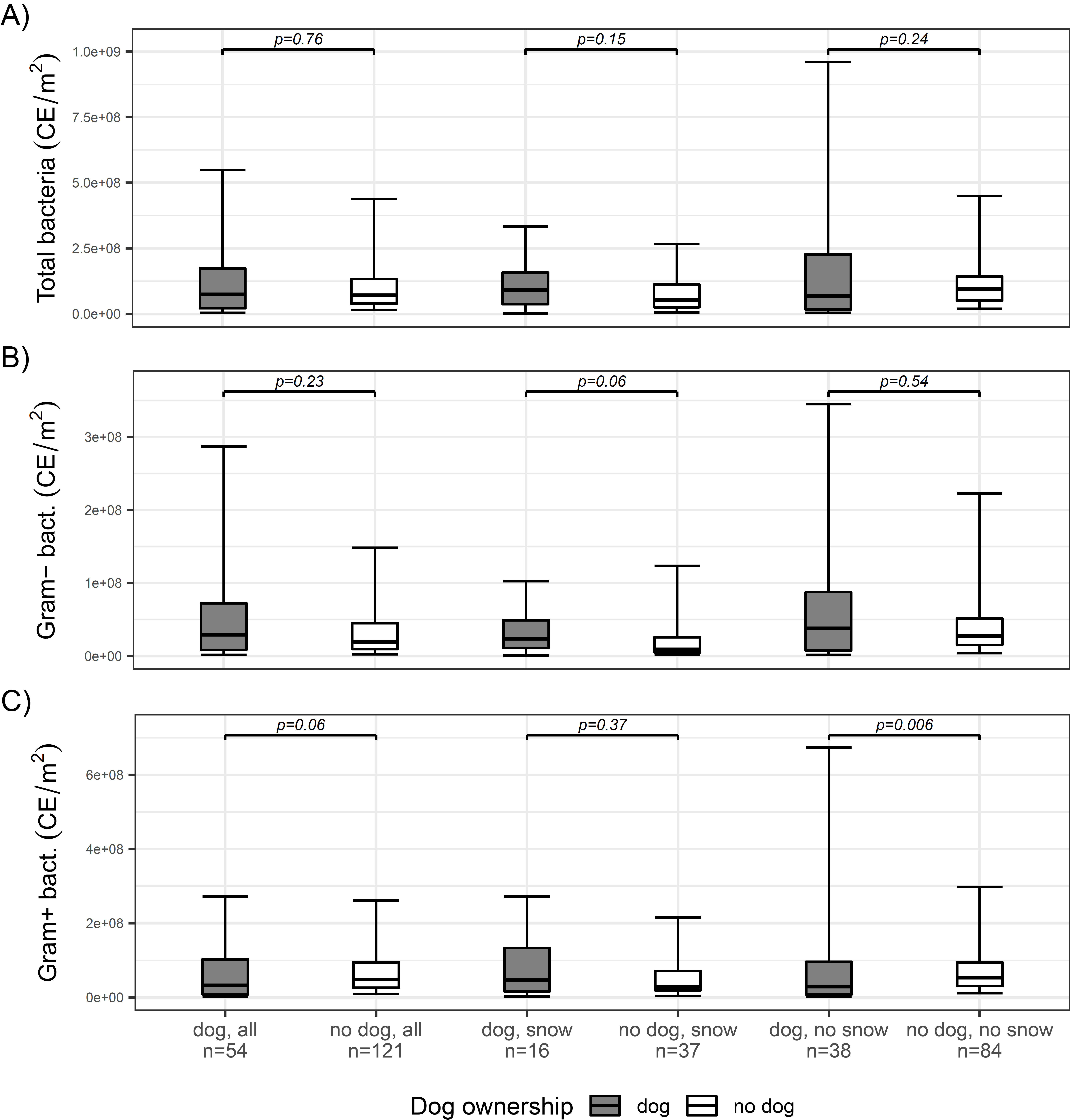


**Additional** Figure S7**.** Box-plots of the bacterial qPCR results (loads) in LUKAS2, stratified by snow cover. Box-plots of the levels of loads (cell equivalent [CE] per square meter sampled floor area) of quantitative polymerase chain reaction (qPCR): A) total bacteria, B) Gram-negative (Gram- bact.) and C) Gram-positive (Gram+ bact.) bacteria in house dust samples collected from dog homes (grey) and non-dog homes (white) in LUKAS2 (all), and stratified by snow cover (snow=dust samples collected when snow covered the ground; no snow=collected without snow cover). P-values from Mann-Whitney U-test. The boxplots present 5^th^ percentile, first quartile, median, third quartile, and 95^th^ percentile of the values.


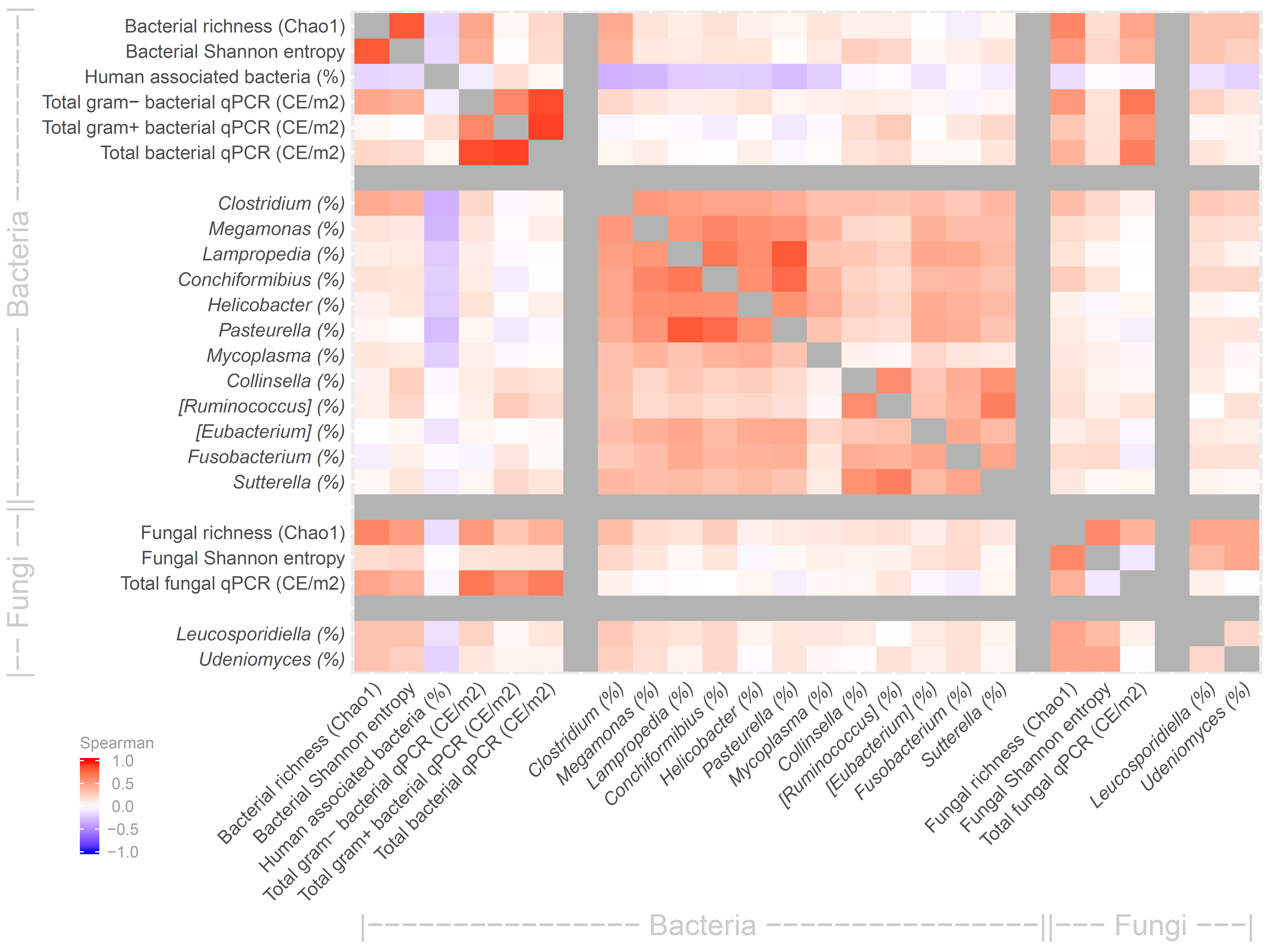


**Additional** Figure S8**.** Heat map of Spearman correlations between bacterial and fungal markers or genera in LUKAS2 cohort. Heat map was created using R software. (%) defines relative abundance; qPCR quantitative polymerase chain reaction; CE cell equivalent; m2 per square meter sampled floor area; brackets indicate candidate taxonomy.
